# Nanoreactor‐Driven Uniform Nano ZnS Deposition in Tunable Porous Carbon Spheres for High‐Performance Zn‐S Batteries

**DOI:** 10.1002/advs.202505218

**Published:** 2025-05-08

**Authors:** Yuxuan Jiang, Bingxin Sun, Dan Wang, Yan Yan, Mohsen Shakouri, Han‐Yi Chen, Wang Zhang, Rongmei Zhu, Huan Pang

**Affiliations:** ^1^ School of Chemistry and Chemical Engineering Yangzhou University Yangzhou Jiangsu 225009 P. R. China; ^2^ Canadian Light Source University of Saskatchewan Saskatoon Saskatchewan S7N 2V3 Canada; ^3^ Department of Materials Science and Engineering National Tsing Hua University 101, Sec. 2, Kuang‐Fu Road Hsinchu 300044 Taiwan; ^4^ State Key Laboratory of Coordination Chemistry Nanjing University Nanjing 210093 P. R. China

**Keywords:** hierarchical porous carbon spheres, nanoreactor, uniform ZnS deposition, Zn‐S batteries

## Abstract

Zn‐S batteries have garnered widespread attention in recent years due to their higher safety and low cost. However, challenges such as incomplete sulfur redox reactions and the tendency of ZnS to agglomerate have impeded the continued advancement of high‐performance Zn‐S batteries. Hollow hierarchical porous carbon spheres (HCs) are designed as efficient sulfur hosts for Zn‐S batteries. The tailored HCs, featuring optimized shell thickness, hierarchical porosity, facilitate uniform nano‐ZnS deposition, and improve ion/electron transport, which are revealed by in situ impedance technology. This nano reactor design ensures highly reversible S‐ZnS conversion, reducing internal polarization and mitigating structural degradation. Electrochemical tests demonstrate outstanding cycling stability, with minimal capacity decay (0.068%) over 500 cycles, and 463 mAh g^−1^ reversible capacity at 5 A g^−1^. Finite element simulations further confirm the effective stress dispersion of HCs, preserving electrode integrity. This work provides a promising strategy for developing high‐performance Zn‐S batteries.

## Graphical abstract

In this work, a series of porous carbon spheres with tunable pore size, particle size, and shell thickness are designed as nanoreactors for the S‐ZnS redox reactions in Zn‐S batteries. The presence of abundant defects and oxygen‐containing functional groups facilitates efficient ion/electron transport throughout the reaction process, significantly reducing internal polarization within the battery. The hierarchical microporous‐mesoporous carbon spheres, acting as nanoreactors, effectively suppress the agglomeration of S and ZnS during cycling, thereby enhancing the redox efficiency of sulfur and improving overall battery performance.

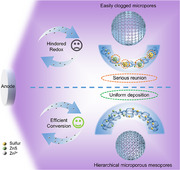

## 1 Introduction

Traditional slfur‐based batteries, such as lithium‐sulfur (Li‐S) and sodium‐sulfur (Na‐S) batteries, have gained popularity in recent years due to their extraordinarily high theoretical capacities and inexpensive cost.^[^ ^1^, ^2^, ^3^ ^]^ These battery technologies have prompted much study into overcoming the inherent problems of sulfur cathodes, such as low electrical conductivity, the dissolution of intermediate polysulfides, and significant volume growth during cycling.^[^ ^4^, ^5^ ^]^ Despite significant breakthroughs in Li‐S and Na‐S batteries, concerns such as the shuttle effect in Li‐S systems and the high operating temperatures necessary for Na‐S batteries have motivated research into alternate systems.^[^ ^6^, ^7^, ^8^ ^]^ In this regard, Zn‐S batteries have emerged as a viable energy storage technology. Zinc, with its abundant availability, low cost and low redox potential, is an attractive anode material for a wide range of aqueous batteries, while sulfur is known for its very high theoretical capacity and environmental friendliness.^[^ ^9^ ^]^ Despite these inherent advantages, the realization of high‐performance Zn‐S batteries remains hampered by critical challenges.^[^ ^10^ ^]^ The naturally insulating nature of sulfur, coupled with its pronounced volume expansion during cycling and the tendency of both elemental sulfur and its discharge product, ZnS, to aggregate, combine to inhibit fast redox kinetics and robust cycling stability.^[^ ^11^ ^]^ To mitigate these problems, the predominant strategy has been to incorporate conductive carbon‐based materials that not only enhance the limited conductivity of sulfur by providing extensive electron pathways, but also act as porous hosts to inhibit ZnS aggregation. Zhu et al. used carbon nanotubes as sulfur hosts to achieve a low voltage hysteresis of 0.47V,^[^ ^12^ ^]^ while Zhi et al. used carbon cloth as a current collector and copolymer cathode to achieve stable cycling at a current of 1A g^−1^.^[^ ^13^ ^]^ Although such approaches have yielded significant improvements in sulfur cathode performance by counteracting the adverse effects of volumetric changes, the isolated benefits of enhanced conductivity and suppressed particle aggregation remain insufficient to fully address the complex challenges presented by Zn‐S systems.

Hollow hierarchical porous carbon spheres, which have emerged as one of the most promising integrated material systems, have been designed to act both as nano‐reactors for sulfur redox reactions and as templates to prevent aggregation of sulfur.^[^ ^14^, ^15^ ^]^ Their hollow interiors, which provide ample space to accommodate sulfur, combined with a hierarchical pore network comprising both micropores and mesopores that facilitate rapid ion diffusion and ensure uniform distribution of reaction products, constitute a unique nano reactor architecture.^[^ ^16^ ^]^ The tailored shell thickness of these carbon spheres not only helps to improve electrical conductivity by creating efficient electron transport pathways but also serves to mitigate the mechanical stresses induced by the significant volume expansion of sulfur during cycling. Constrained by the inherent nanoconfinement effect, the growth of ZnS particles is maintained at the nanoscale, thereby maintaining a high active surface area and ensuring fast, reversible redox kinetics. Enabled by the nano reactor effect, uniform nano‐ZnS deposition is achieved, minimizing the detrimental effect of particle aggregation on both the overall reaction rate and the cycling stability of the battery. In addition, the incorporation of oxygen‐containing functional groups on the carbon surface further enhances electrolyte wettability and provides additional active sites for the sulfur redox process, reducing internal polarization and improving overall electrochemical performance.

In this regard, we have conveniently synthesized defect‐rich hollow conductive carbon spheres with adjustable particle and pore sizes as the sulfur hosts for Zn‐S batteries by changing the silicon source, and a series of in situ/ex situ tests have demonstrated the superiority of the hollow hierarchical porous carbon spheres as the “nano‐reactors” of Zn‐S batteries, and furthermore, through the characterization of the electrode during the reaction process, it is demonstrated that the carbon sphere template can be used as a template for the reversible redox reaction of S‐ZnS, which effectively improves the agglomeration of active substances and promotes the efficient and reversible reaction. This integrated strategy, namely enhancing the electrical conductivity by optimizing the carbon structure, limiting the ZnS growth by effective nanoconfinement, and relieving stresses during volume changes by fine‐tuning the shell thickness and pore size distribution, provides an interesting idea for the design of high‐performance and durable Zn‐S batteries.

## 2 Results and Discussion

**Figure 1:**
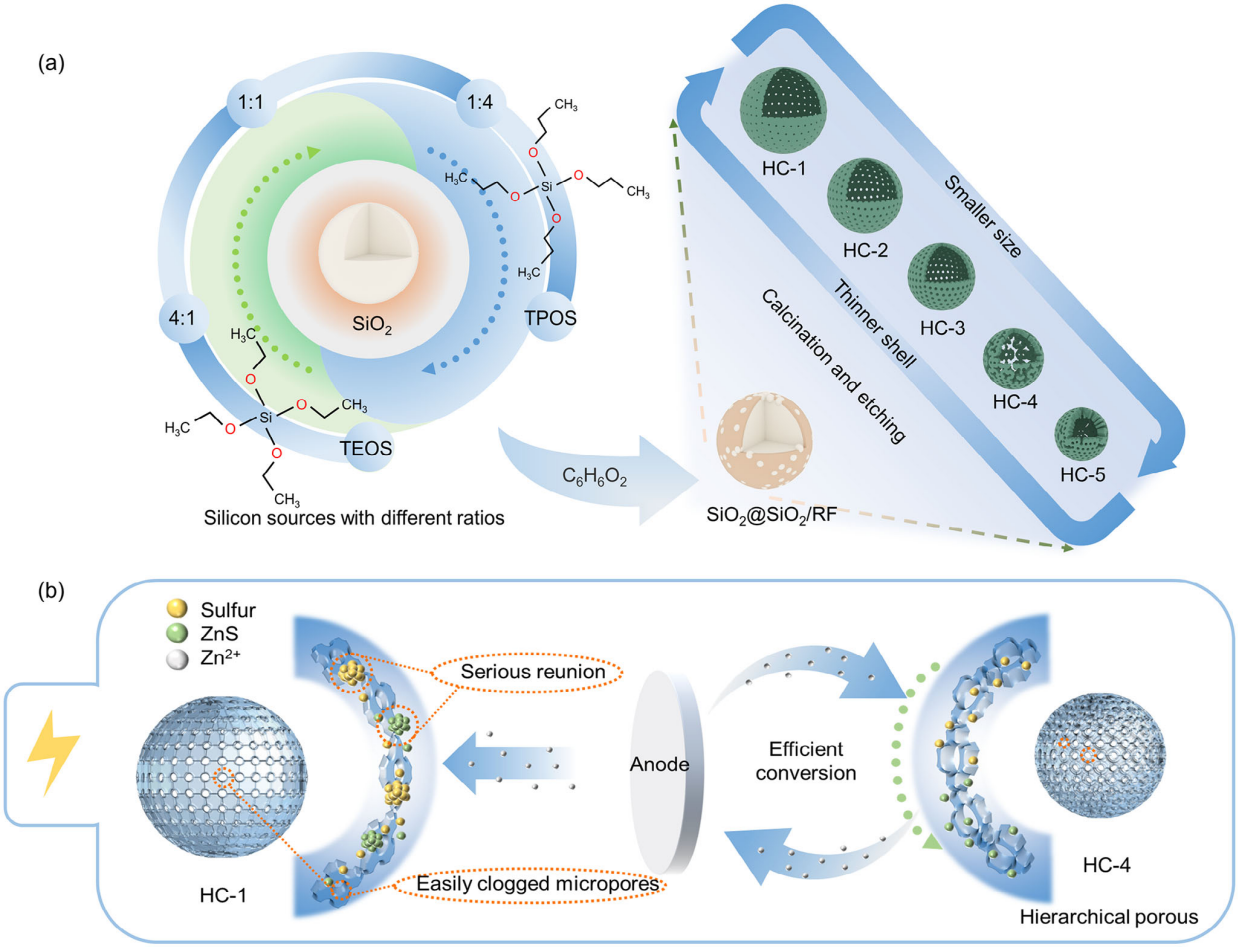
a) Schematic of the preparation of porous carbon spheres with different sizes and shell thicknesses; b) Mechanism by which HCs improve the performance of Zn‐S batteries.

**Figure** **1**a presents the synthetic strategy for fabricating hollow carbon spheres with tunable morphologies and structural features by varying the ratio of silicon precursors, TEOS (tetraethyl orthosilicate) and TPOS (tetrapropyl orthosilicate). Through the controlled hydrolysis‐condensation of these precursors, silica spheres (SiO_2_) with tailored particle sizes and shell properties were obtained. These SiO_2_ spheres were then utilized as hard templates for the uniform deposition of a resorcinol‐formaldehyde resin layer (SiO_2_@SiO_2_/RF) via polycondensation under alkaline conditions. Following calcination at 700°C in N_2_ for 5 h, SiO_2_@SiO_2_@C composites were produced. The SiO_2_ templates were subsequently removed using NaOH solution, resulting in HCs with adjustable structural characteristics. Figure 1b demonstrates the mechanistic of HCs as nanoreactors to promote the uniform deposition of ZnS as well as the efficient oxidative reduction of sulfur.

**Figure 2:**
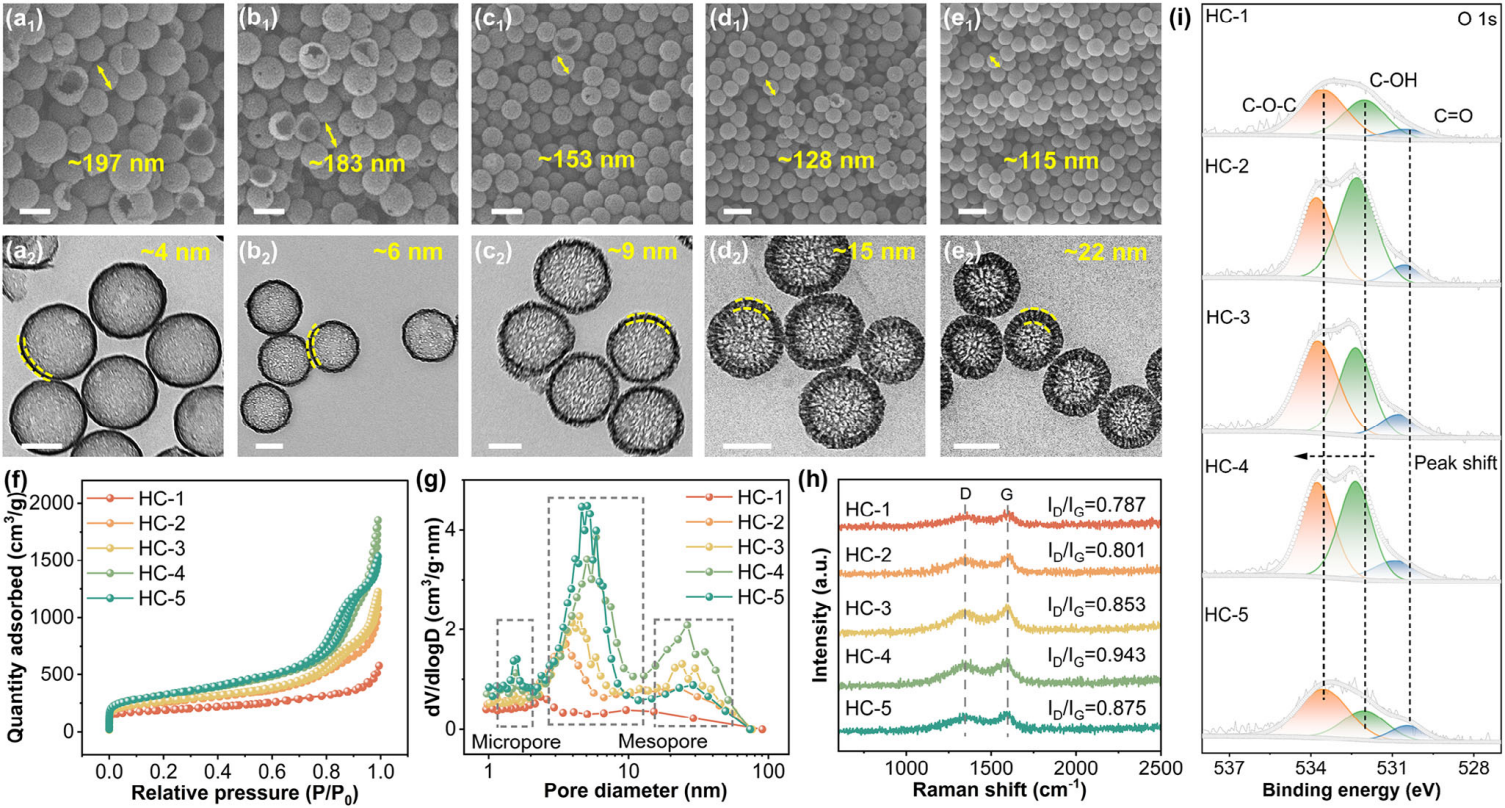
Characterization of porous carbon spheres (HC‐1/HC‐2/HC‐3/HC‐4/HC‐5): a_1_–e_1_): SEM image; a_2_–e_2_): TEM image; f) Nitrogen adsorption isotherms and g) pore size distribution of different carbon spheres; h) Raman spectroscopy; i) XPS spectra of O 1s. The scale bars in the graphs are 200 nm (a_1_–e_1_) and 100 nm (a_2_–e_2_).

As the TPOS ratio increased, the carbon spheres exhibited a gradual reduction in particle size along with thicker shells. These structural and morphological changes are comprehensively analyzed in **Figures** **2** and S1 (Supporting Information) using scanning electron microscopy (SEM), transmission electron microscopy (TEM), X‐ray diffraction (XRD), and related characterization methods. Figure 2a–e capture how the structure of the hollow carbon spheres evolves as the ratio of TEOS to TPOS changes. When TEOS is the only silicon source (HC‐1, Figure 2a_1_,a_2_), the resulting spheres are ∼200 nm in diameter, with a tightly packed shell ≈4 nm. As the proportion of TPOS increases (Figure 2b–e), the spheres shrink in size, eventually reaching about 118 nm, while their shells grow noticeably thicker, peaking at around 22 nm when TPOS is used exclusively. Alongside these changes, the decomposition behavior of the silica‐oxygen network and its accompanying gas release are similarly modulated by the silicon source ratio, with a high TPOS ratio contributing to the introduction of more pores the initially dense shells gradually become more porous as TPOS takes over.

This evolution stems from the contrasting behaviors of TEOS and TPOS during hydrolysis and condensation. TPOS polymerizes at a slower rate than TEOS in the reaction system since propoxy has a greater steric impact than ethoxy, SiO_2_ particles are continually and slowly released, and they evenly nucleate and self‐assemble to create SiO_2_ core particles that serve as templates for the cavities. When RF begins to polymerize and nucleate on the surface of SiO_2_ core particles, a high number of SiO_2_ particles can coexist with them. SiO_2_ particles and aggregates engage with RF oligomers via weak hydrogen bonds before aggregating onto the SiO_2_ core to produce the SiO_2_@SiO_2_/RF core‐shell structure.^[^ ^17^ ^]^ At the same time, the loosely packed silica framework created with TPOS introduces more porosity during the carbonization and etching stages. The XRD pattern shows that all HCs have distinct carbon characteristic peaks (Figure S2, Supporting Information). Figure 2f,g showcase the nitrogen adsorption‐desorption isotherms and pore size distributions of HCs synthesized using varying TEOS/TPOS ratios. In Figure 2f, samples HC‐2 to HC‐5 display evident hysteresis loops, indicative of mesoporous structures formed by capillary condensation within the interconnected network of pores. Among these, HC‐4 shows the highest nitrogen adsorption capacity, reflecting its superior specific surface area and well‐developed porosity. This trend underscores the influence of TPOS content on structural evolution: higher TPOS ratios not only lead to smaller particle sizes and thicker shells but also facilitate the formation of a highly porous carbon framework.

The pore size distribution curves in Figure 2g further detail these structural differences. HC‐1, synthesized exclusively with TEOS, exhibits a compact shell structure and a narrow pore size distribution dominated by micropores with only a minor fraction of mesopores. With the increase in TPOS content, the pore volume fraction of 8 nm and 20 nm pores within the mesoporous structures of HC‐3 to HC‐5 significantly increases. Notably, HC‐4 exhibits the highest pore volume fraction at 20 nm among all samples.^[^ ^18^ ^]^ The hierarchical pore structure of HC‐4, characterized by significant contributions from mesopores and micropores, plays a crucial role in achieving a functional balance critical for sulfur storage, ion and electron transport, and electrolyte accessibility. The hierarchical mesoporous structure facilitates effective electrolyte penetration and provide ample space for sulfur loading, while the micropores enhance sulfur confinement. Additionally, the increased shell thickness in HC‐4, compared to samples synthesized with lower TPOS ratios, improves the structural integrity and electronic conductivity of the carbon spheres, creating more efficient pathways for electron and ion transport.

The Raman spectroscopy analysis of the composite materials provided insights into their defect levels and degree of disorder. Figure 2h displays the Raman spectra of HCs synthesized with different TEOS/TPOS ratios, all of which exhibit the characteristic D and G bands of carbon materials, located at 1345 cm^−1^ and 1584 cm^−1^, respectively. The D band arises from the disorder and structural defects in the carbon framework, serving as an indicator of the degree of disorder‐the stronger the D band, the higher the defect density. The G band, originating from the in‐plane vibrations of sp^2^ hybridized carbon atoms, reflects the degree of graphitization in the carbon material. The defect level is typically quantified by the intensity ratio of the D and G bands (I_D_/I_G_). The I_D_/I_G_ ratios of HC‐1 to HC‐5 are 0.787, 0.801, 0.853, 0.943, and 0.875, respectively. When both TEOS and TPOS are employed as silicon sources, the I_D_/I_G_ ratio increases with the TPOS content, peaking at 0.943 for HC‐4. This elevated I_D_/I_G_ ratio suggests a higher density of disordered structures and edge defects, which can act as active sites for reactions,^[^ ^18^ ^]^ thereby accelerating Zn^2+^ insertion/extraction and enhancing the reversible transformation of S‐ZnS during electrocatalysis.

Complementing the Raman analysis, XPS measurements were conducted to investigate the composition of the HCs in greater detail. Figure S3 (Supporting Information) reveals that all HCs contain abundant C and O elements, with the detected N content likely stemming from the use of ammonia during synthesis and subsequent high‐temperature calcination under an N_2_ atmosphere. The high‐resolution XPS C1s spectrum (Figure S4, Supporting Information) identifies characteristic peaks corresponding to C═C, C═C, and O═C─O bonds at 284.8 eV, 286.3 eV, and 288.9 eV respectively.^[^ ^19^ ^]^ The N1s spectrum (Figure S5, Supporting Information) is deconvoluted into four peaks at 398.1 eV (pyridinic‐N), 400.2 eV (pyrrolic‐N), 401.4 eV (graphitic‐N), and 403.1 eV (oxidized‐N).^[^ ^7^, ^18^ ^]^ The O 1s spectrum (Figure 2i) further highlights the oxygen functionalities present on the HCs. For HC‐1 and HC‐5, synthesized with single silicon sources, typical peaks corresponding to C = O (530.84 eV), C‐OH (532.28 eV), and C‐O‐C (533.67 eV) are observed.^[^ ^20^ ^]^ As the TEOS/TPOS ratio increases, a progressive shift in the XPS peaks toward higher binding energies is noted, indicating an increase in oxygen‐containing groups on the carbon sphere surface.^[^ ^21^ ^]^ This enrichment of oxygen functionalities plays a pivotal role in enhancing the reversible conversion of S‐ZnS in Zn‐S batteries. The introduction of oxygen groups provides additional active sites for breaking the Zn‐S bonds in ZnS, effectively reducing the bond dissociation energy through localized interactions with Zn atoms, which promotes the S‐ZnS conversion reactions, significantly improving the electrochemical kinetics. Moreover, the presence of oxygen‐containing groups enhances the wettability of the carbon sphere surface, facilitating better electrolyte penetration and optimizing the transport pathways for Zn^2+^ and S^2−^ at the electrode‐electrolyte interface. This reduces interfacial resistance and further boosts the reaction efficiency and cycle stability of the battery.

**Figure 3:**
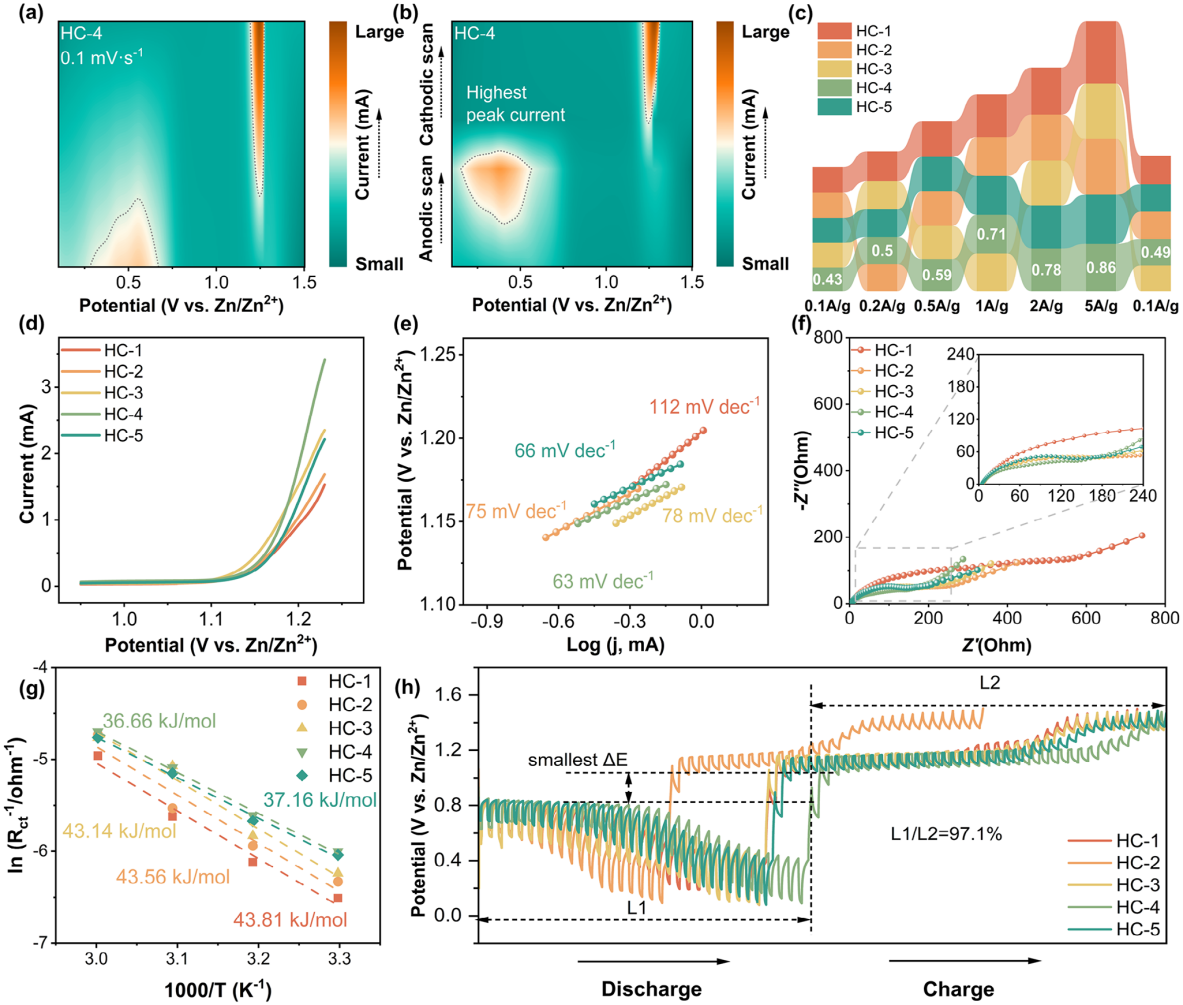
CV curves of HC‐4 at a) 0.1 mV s^−1^ and b) different scan rate; c) The delta E values of cells with various carbon spheres at different currents; d) Extracted parts of the CV curves in (Figure S7, Supporting Information) between 0.1 and 1.5 V and e)Tafel plots of Peak A;f) Nyquist curves and g) Arrhenius plots based on charge transfer resistance of batteries with HCs; h) GITT curves.

To evaluate the performance of different HCs as sulfur hosts during battery cycling, a series of electrochemical performance tests were conducted. **Figures** **3**a and S6 (Supporting Information) present contour plots of cyclic voltammetry (CV) curves for different HCs, while Figure S7 (Supporting Information) shows the corresponding raw CV curves. From the contour plots, it is evident that HC‐1 exhibits the smallest peak current (Figure S6a, Supporting Information). As the TEOS/TPOS ratio increases, electrode reactions become more complete. Among all samples, HC‐4, with its abundant defect sites and oxygen‐containing functional groups, demonstrates the best reversibility during cycling. At a sweep rate of 0.1 mV s^−1^, its CV curves over three cycles nearly overlap (Figure S7d, Supporting Information), showcasing minimal polarization and the strongest peak current. The CV curves at different scanning rates are shown in Figure 2b and Figures S8 and S9 (Supporting Information), under varying sweep rates (Figure 2b; Figure S9d, Supporting Information), the CV curves of HC‐4 maintain distinct redox peaks and low polarization, indicating that its hierarchical porous structure effectively facilitates the S‐ZnS reaction kinetics. The rate performance tests (Figure S10, Supporting Information) further support this observation. As the current density increases from 0.1 A/g to 5 A/g, the capacity of HC‐1 decreases sharply. This decline is likely due to the inability of its thinner carbon shell to accommodate the significant volume changes of sulfur during charge‐discharge cycles, leading to shell rupture and rapid performance degradation. In stark contrast, HC‐4 retains a capacity of 463 mAh g^−1^ at 5 A g^−1^. Its relatively thicker shell better tolerates the volume expansion of sulfur, while its smaller particle size enhances contact with the electrolyte, thereby improving reaction kinetics at high currents.

Figure S11 (Supporting Information) showcases the charge‐discharge curves of different HCs at 0.1 A/g. All batteries exhibit a small plateau at approximately 1.3 V, attributed to the redox reaction involving the ZnI_2_ additive in the electrolyte, as confirmed by the iodine‐related redox peaks in the CV curves (Figure S7, Supporting Information). At lower voltages, a longer discharge plateau at 0.7 V is observed across all cells, but the plateau for HC‐1 and HC‐2 declines rapidly. This can be attributed to their larger particle sizes, which hinder sufficient contact between active materials and the electrolyte. In contrast, HC‐4 benefits from its smaller particle size and optimized carbon shell thickness, achieving superior sulfur utilization and lower internal polarization (delta E). Galvanostatic charge‐discharge (GCD) test on sulfur‐free cathode batteries also show that ZnI_2_ as an electrolyte additive provides almost no additional capacity (Figure S12, Supporting Information). The polarization behavior observed in rate performance tests is consistent with that under low current densities (Figure S11, Supporting Information). Figure 3c showcases the charge‐discharge polarization of different HCs at varying current densities. HC‐4 exhibits the smallest polarization, highlighting how its hierarchical porous structure‐combining micropores and mesopores‐provides efficient transport channels for Zn^2+^ and S^2−^. Additionally, the introduction of oxygen‐containing functional groups enhances electrolyte wettability, ensuring better interface contact and facilitating ion transport. This improved interfacial contact and ionic pathway accelerates charge transfer at the interface, mitigating concentration polarization under high current densities. Furthermore, the interconnected conductive grooves on HC‐4's surface disperse electron transport pathways, preventing localized current concentration and reducing ohmic polarization. This uniform electron distribution significantly enhances the overall reaction activity of the electrode.

To further investigate the impact of different HCs on battery kinetics, portions of the CV curves were extracted (Figure 3d) to calculate Tafel slopes (Figure 3e). HC‐4 demonstrates the fastest S‐ZnS conversion kinetics, as evidenced by its lower Tafel slope. A smaller Tafel slope indicates stronger charge transfer capabilities during the redox process. Figure 3f,g show the electrochemical impedance spectroscopy (EIS) of different HCs at room temperature and the activation energy of charge transfer reactions at different temperatures, with the corresponding equivalent circuit fitting in Figure S13 (Supporting Information). Owing to its smaller particle size, HC‐4 exhibits reduced solution diffusion resistance and charge transfer resistance (*R*_ct_). The abundant defects in HC‐4 enhance reaction kinetics across different temperatures. Using the Arrhenius equation, the diffusion activation energy (*E*_a_) for charge transfer was determined. HC‐4 has an *E*_a_ of 30.7 kJ mol^−1^, lower than HC‐5's 31.2 kJ mol^−1^, indicating stronger charge transfer capabilities and faster S‐ZnS conversion kinetics.^[^ ^22^ ^]^ Lower activation energy typically corresponds to reduced interfacial and ionic diffusion resistance, reflecting more efficient electrochemical reactions. Galvanostatic Intermittent Titration Technique (GITT) testing (Figure 3h) further reveals the reaction kinetics of the electrode materials by analyzing potential responses and polarization behavior.^[^ ^23^ ^]^ HC‐4 exhibits smaller polarization, indicating a lower energy barrier for S‐ZnS conversion and a more reversible reaction pathway. This suggests that the electrode material can balance the generation and consumption of active species during charge and discharge cycles, maintaining excellent cycling stability. Lower polarization also reflects a significant reduction in interfacial impedance. HC‐4's hierarchical porous structure and surface functionalization facilitate rapid Zn^2+^ and S^2−^ transport, optimizing ion exchange efficiency between the electrolyte and electrode. Moreover, the closer ratio of discharge to charge capacities (L1/L2) to 100% indicates a stable ion and electron transport network and higher active material utilization throughout the reaction process. The oxygen‐containing functional groups or defect sites on HC‐4's surface catalyze Zn─S bond cleavage and stabilize reaction intermediates, effectively enhancing S‐ZnS conversion kinetics.

**Figure 4:**
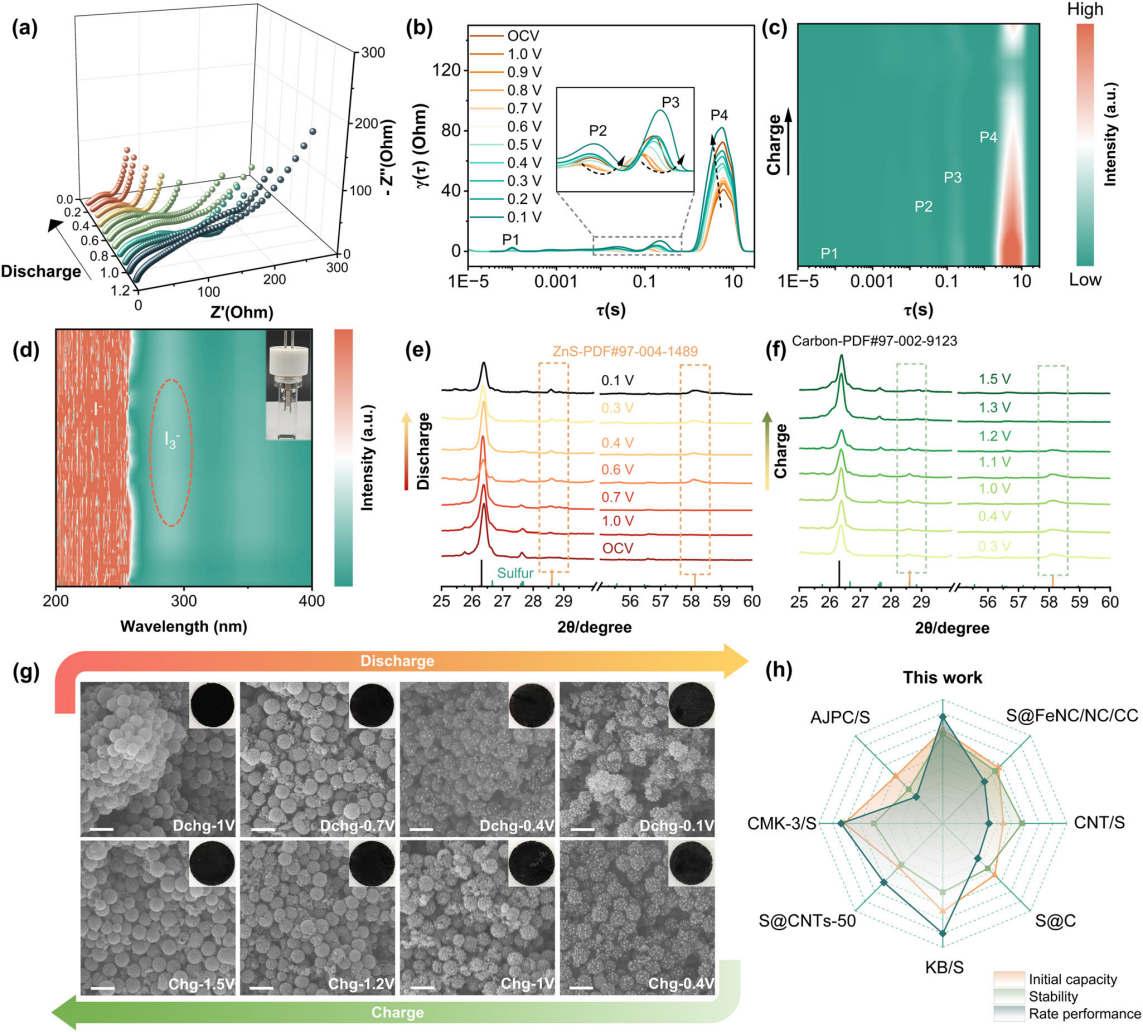
Reaction processes via ex situ/in situ characterization: a) impedance changes during battery discharge and the corresponding b) DRT curves, c) corresponding contour plots of the DRT during charging; d) In situ UV/Vis absorption during the charging phase; XRD patterns of dynamic detection during e) discharging and f) charging of batteries; g) SEM images of cathodes at vary voltages during the cycling process (The scale bars in the graphs are 200 nm). h) Comparison with other reported sulfur hosts for Zn‐S batteries.

Apart from conventional electrochemical tests, we also dynamically monitored the reaction process of the Zn‐S battery by employing ex situ/in situ tests. We employed in situ EIS to investigate the impedance evolution of Zn‐S batteries during cycling, providing insight into the kinetics of electrochemical reactions at the electrode/electrolyte interface. During the initial discharge stage, the semicircle in the mid‐frequency region of the Nyquist plot significantly decreased, indicating a reduction of *R*_ct_. This phenomenon reflects the activation of the electrodes (**Figure** **4**a; Figure S14, Supporting Information) and is closely associated with the electrochemical reaction rate at the interface. Batteries utilizing HC‐1 as the sulfur host exhibited a slower decrease in impedance during discharge, suggesting a relatively low interfacial reaction rate. Additionally, a larger Warburg impedance was observed in the low‐frequency region, indicating significant diffusion resistance for electrolyte ions within the electrode pores. In contrast, the HC‐4 electrode showed a more pronounced decrease in impedance with decreasing voltage, and the slope in the low‐frequency region approached verticality, indicating more rapid Zn^2+^ diffusion from the electrolyte to active sites. This improvement can be attributed to the abundant defect sites, optimal particle size, and shell thickness of HC‐4, which collectively enhance reaction kinetics.

In the mid‐to‐low frequency region, the impedance changes became more complex (Figure 4a; Figure S14, Supporting Information). To accurately analyze these processes, we employed the distribution of relaxation times (DRT) method. The DRT spectra revealed four distinct peaks (Figure 4b; Figure S15, Supporting Information), labeled as P1, P2, P3, and P4. The high‐frequency P1 peak, which reflects the contact resistance at the electrode interface, was minimally affected by the discharge state.^[^ ^24^, ^25^ ^]^ The smaller area of the P1 peak for HC‐4 indicates lower internal contact resistance, attributed to its smaller particle size, which enhances the contact between active materials and the electrolyte, thereby reducing internal resistance. P2 and P3 correspond to ion transport at the anode and cathode interfaces, respectively, while P4 is typically associated with the charge transfer process at the cathode. In Zn‐S batteries, the P4 peak is directly linked to the S‐ZnS conversion reaction, a process whose kinetics are often constrained by interfacial reactions and play a critical role in battery performance. During discharge, the P2 and P3 peaks for the HC‐1 electrode shifted toward higher intensities, indicating localized active reaction regions in ion transport, which result in interfacial reaction non‐uniformity (Figure S15, Supporting Information). Such non‐uniformity may cause stress concentration, reducing electrode structural stability and long‐term cycling performance. Conversely, for HC‐4, the P2 and P3 peaks shifted toward the low‐frequency region, suggesting increased relaxation time constants for ion transport, likely due to the hierarchical porous carbon structure that promotes deeper ion penetration and more uniform distribution (Figure 4b). Although the relaxation time constants increased slightly, the P4 peak shifted to higher frequencies during discharge, indicating an acceleration of the charge transfer process. Moreover, the HC‐4's lower intensity of the P4 peak implies more uniform interfacial reactions, mitigating stress concentration and side reactions, and enhancing long‐term cycling stability.^[^ ^26^ ^]^ This observation aligns with the significantly reduced impedance of the HC‐4 electrode in the Nyquist plot, further demonstrating the superior interfacial stability and reaction efficiency of HC‐4 as a sulfur host in Zn‐S batteries.

During the charging process (Figure 4c; Figure S16, Supporting Information), in situ EIS monitoring of the Zn‐S battery revealed significant advantages of the HC‐4 electrode as a sulfur host. The results showed that the P1 to P3 peaks of the HC‐4 electrode exhibited overall weaker intensities, indicating lower contact resistance and ionic transport resistance. Meanwhile, the P4 peak intensity gradually diminished during charging, with a rapid drop to a notably low level at lower charging voltages. This behavior suggests that the HC‐4 sulfur host effectively accelerates the conversion of ZnS to S, completing this process at lower voltages.^[^ ^27^, ^28^ ^]^ The optimized particle size, shell thickness, and abundant defect sites of HC‐4 collectively enhance the reaction kinetics and interfacial conversion efficiency. In contrast, the HC‐1 electrode's P1 to P3 peaks showed higher intensities, indicating greater interfacial contact resistance and ionic transport resistance. Although the P4 peak intensity also decreased during charging, the voltage required to reduce it to a near‐minimum level was significantly higher than that for the HC‐4 electrode. This indicates that the HC‐1 sulfur host requires a greater driving force to facilitate the ZnS‐to‐sulfur conversion. Furthermore, after full charging, the P4 peak intensity of HC‐1 remained lower than that of HC‐4, suggesting incomplete conversion of ZnS to S. This incomplete conversion not only limits sulfur utilization but also likely results in the formation of inert phases within the electrode, further hindering electrochemical reaction uniformity and stability.

In the in situ UV/Vis absorption of the Zn‐S battery, the absorption peak around 220 nm can be attributed to I^−^, when HC‐4 was used as the sulfur host, the absorption peak of I_3_^−^ ions gradually increased and decreased during charge (Figure 4d).^[^ ^29^, ^30^ ^]^ This result clearly demonstrates that ZnI_2_, as an electrolyte additive, can successfully participate in electrochemical reactions, facilitating the redox process of sulfur, with enhanced reversibility and cycling stability. In addition, ex situ XRD was utilized to validate the reversible transformation of S‐ZnS (Figure 4e,f), with diffraction peaks around 26.4° primarily ascribed to amorphous carbon, and ZnS‐related diffraction peaks emerging at 28.6° and 58.3° as the voltage declines to approximately 0.7 V.^[^ ^31^ ^]^ This outcome corresponds to the discharge plateau observed around 0.7 V in the GCD curves (Figure S11, Supporting Information), and the intensity of the diffraction peaks for ZnS progressively increases. During the charging process, which is congruent with the GCD curve, the S‐ZnS conversion was essentially complete at approximately 1.2–1.3 V, where the ZnS diffraction peaks diminished and eventually became nearly imperceptible, suggesting the near‐complete conversion of ZnS back to S. Meanwhile, the ex situ XPS spectra further demonstrated the reversible transformation of S and ZnS, with the strong peaks at 162.2 eV and 163.3 eV mainly belonging to S‐Zn and the peaks of S‐S/C‐S‐C barely visible at discharge to 0.1 V (Figure S17, Supporting Information).

Additionally, we conducted detailed SEM analyses to monitor the morphological evolution of the cathode during the charge and discharge processes of the Zn‐S battery (Figure 4g), aiming to better understand the dynamic behavior of carbon spheres and surface deposition. During OCV discharge to 1V, the carbon spheres retained their initial porous structure without notable changes, and the optical appearance of the electrode remained like that of the pristine electrode. However, as the voltage decreased further, small nano‐sized particles began to appear on the surface of the carbon spheres, gradually shifting the electrode color from deep black to a slightly grayish white as ZnS formed. Coupled with ex situ XRD results (Figure 4e), we hypothesize that nano ZnS deposition began to occur on both the internal and external surfaces of the carbon spheres at this stage, corresponding to approximately 0.7V. This observation aligns well with the turning point in the GCD curve (Figure S11, Supporting Information). As discharge progressed, the density of nano ZnS particles on the carbon sphere surfaces increased, reaching a peak at 0.1V. At this point, the XRD data proved that most of the sulfur was involved in the reaction and was successfully converted into the discharge product ZnS, and the electrode's optical image transitioned from its initial black to a deep gray (Figure 4g), reflecting the uniform deposition of ZnS on the cathode surface. Upon switching to the charging process, the electrode's deep gray appearance gradually reverted to its original black as the voltage increased. SEM images revealed that the nano ZnS particles on the carbon sphere surfaces diminished progressively. By 1.2V, the surface particles had become sparse, and by 1.5V, the particles were nearly absent. Notably, the carbon spheres retained their original porous structure without any visible cracks or degradation. These findings are consistent with the results shown in Figure 4f. Through these characterization results, it can be clearly seen that the HCs acted as spherical templates, effectively promoting the uniform dispersion and deposition of ZnS nanoparticles while maintaining their structural completeness. Its unique porous structure and physical stability lay the foundation for achieving the long‐cycle performance of ZnS batteries. The graded porous carbon spheres also demonstrated excellent electrochemical performance in comparison with other carbon‐based sulfur hosts (Figure 4h and Table S1, Supporting Information).

**Figure 5:**
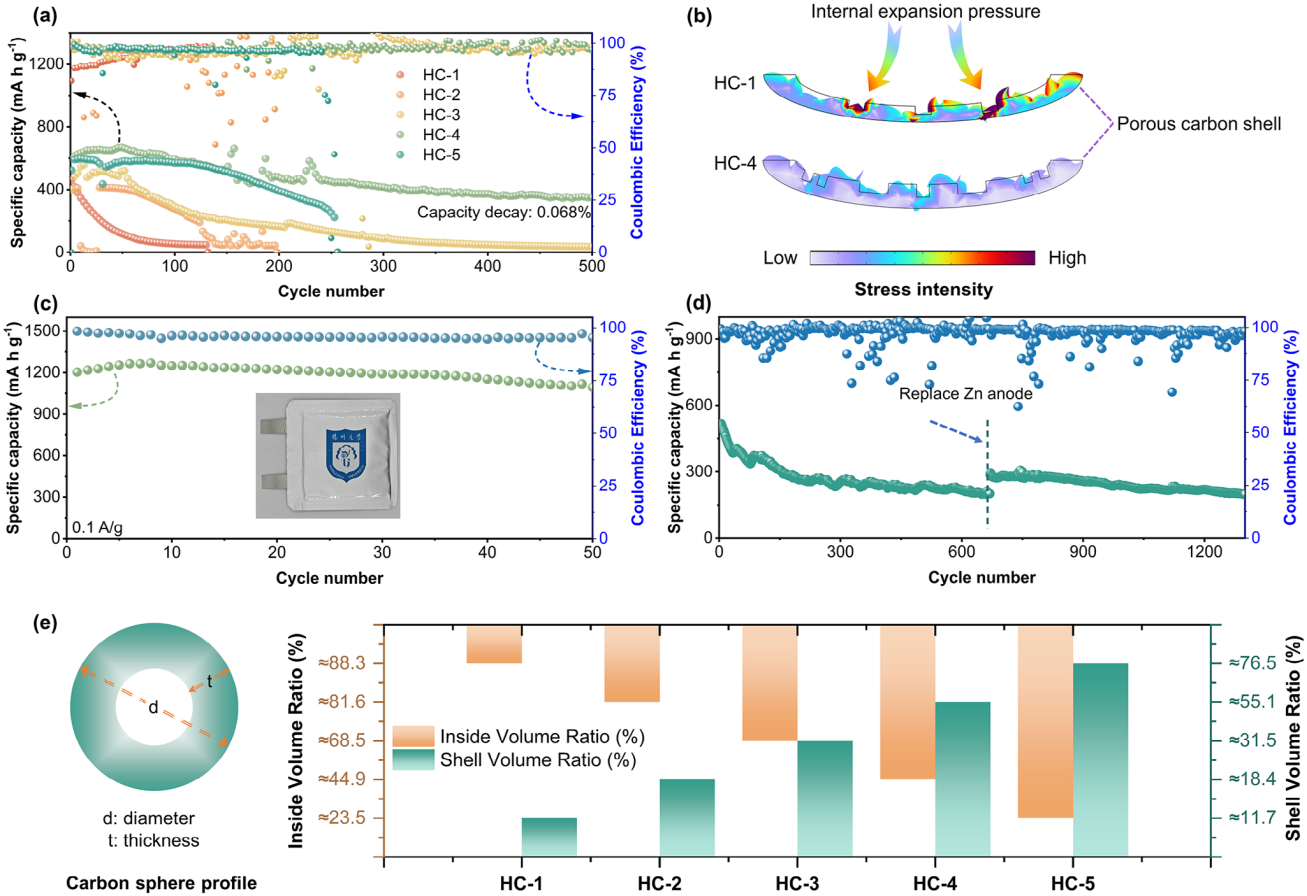
a) Performance of HCs over long cycles, b) stress distribution at the HCs shell, c) cycling performance of pouch batteries, d) effect of replacement of anode on the cycling stability of batteries e) the volume share and shell share of different carbon spheres.

The long‐term cycling performance of Zn‐S batteries with different carbon spheres is compared in **Figure** **5**a. HC‐1 demonstrates inferior cycling stability due to its structural limitations. With fewer defects and larger particle size, HC‐1 exhibits poor electrical conductivity, which hinders the efficient transport of ions and electrons. This restricted transport capability exacerbates internal resistance and leads to rapid capacity fading during prolonged cycling. In contrast, HC‐4 achieves exceptional cycling stability, maintaining a capacity decay rate as low as 0.068% after 500 cycles at 1A/g. This outstanding performance is attributed to HC‐4's smaller particle size and optimized shell thickness, which enhance electrical conductivity and reduce internal polarization. Moreover, the abundant surface defects and oxygen‐containing functional groups on HC‐4 facilitate the reversible S‐ZnS conversion process and improve electrolyte wettability, creating a more stable electrochemical environment for long‐term operation. Figure 5b further elucidates the impact of carbon sphere structures on battery performance through finite element simulations. Models of HC‐1 and HC‐4 carbon sphere shells were constructed, and compressive forces were applied to the inner surfaces to simulate the stress generated by the volumetric expansion of sulfur during cycling. The stress distribution across the HC‐1 shell model reveals significant stress concentration around the pore regions. Due to the sparse distribution of its pores, HC‐1 struggles to effectively alleviate the stress caused by sulfur expansion, which likely contributes to its rapid capacity decay over extended cycles. In contrast, HC‐4, with its hierarchical microporous‐mesoporous structure and denser pore distribution, effectively disperses stress throughout the shell during cycling. This structural advantage mitigates the impact of volumetric expansion, enabling HC‐4 to maintain superior cycling stability. These findings underscore the critical role of rational structural design in achieving high‐performance Zn‐S batteries. The practical application of the pouch cell is shown in Figure S18 (Supporting Information), which still achieves a Coulombic efficiency of 98. 4% at high loads (Figure 5c). This outstanding performance, attributed to the HC‐4 cathode, was further confirmed by the corresponding GCD curves (Figure S19, Supporting Information). To investigate the capacity fading mechanism in Zn‐S batteries, further analysis was performed using Swagelok cells (Figure 5d). After approximately 700 cycles at a current density of 2A/g, the battery exhibited noticeable capacity fading. Upon replacing the Zn anode, the cell showed a slight capacity recovery and maintained a high‐capacity retention over an additional 600 cycles. These observations indicate that the adverse effects of side reactions on the Zn anode, such as dendrite growth or by‐product accumulation, were relatively minor compared to the degradation on the sulfur cathode.^[^ ^10^ ^]^

Figure 5e illustrates the volume share and shell share of different carbon spheres (specific data in Table S2, Supporting Information), the superiority of HC‐4 can be understood from both dimensional and volumetric perspectives. Although HC‐1, boasting a core volume ratio of approximately 88.3%, might initially appear advantageous for hosting sulfur, its extremely thin shell‐constituting only about 11.7% of the total volume—fails to provide adequate confinement. While such a minimal shell thickness may offer a low diffusion barrier, it unfortunately results in poor control over ZnS aggregation, thereby adversely affecting the reversibility of sulfur redox reactions. On the other hand, HC‐5, characterized by a very thick shell that occupies roughly 76.5% of the total volume, impedes Zn ion diffusion, as the diffusion time is roughly proportional to the square of the shell thickness (τ ∝ t^2^/D, where D represents the diffusion coefficient). Although this robust shell imparts excellent mechanical stability, it creates a substantial diffusion barrier, ultimately reducing the overall reaction rate.

HC‐4, with a core volume ratio of about 44.9% and a shell volume fraction of approximately 55.1%, achieves an optimal balance between providing ample active sites and maintaining a sufficiently short diffusion path. In terms of the dimensionless ratio (d – 2t)/t, HC‐4 registers a value of approximately 98/15, or about 6.5, which signifies a condition wherein the shell is thick enough to serve effectively as a nanoreactor—confining the growth of ZnS particles and ensuring uniform nano‐ZnS deposition—yet remains sufficiently thin to allow rapid Zn^2+^ diffusion. This balance is crucial because, as the nanoconfinement effect restricts ZnS growth to the nanoscale, an appropriately moderate shell thickness ensures that the active sulfur, loaded into the carbon sphere via melt infusion, can efficiently react with Zn ions diffusing from the external environment. Consequently, the controlled nanoreactor environment provided by HC‐4 minimizes internal polarization and promotes fast, reversible S–ZnS redox kinetics.

Overall, the remarkable improvement in Zn‐S battery performance can be attributed to the enhanced sulfur cathode kinetics. The hierarchical porous HCs serve as an ideal host for sulfur reactions, offering distinct structural advantages.

Their reduced particle size effectively minimizes internal polarization, thereby improving the uniformity of electrochemical reactions. Furthermore, the synergistic interaction of micropores and mesopores, along with abundant defect sites, provides numerous active sites for the reversible conversion of nano ZnS, significantly enhancing the cycling stability between sulfur and ZnS. At the same time, the appropriately engineered shell thickness not only alleviates structural stress caused by volume expansion during prolonged cycling but also ensures the mechanical stability of the porous carbon spheres. These structural optimizations collectively enhance the utilization of sulfur active material and significantly extend the cycling life of Zn‐S batteries, highlighting the potential of hierarchical porous HCs for high‐performance energy storage applications.

## 3 Conclusion

In summary, our study has demonstrated that the design of hollow hierarchical porous carbon spheres provides an effective strategy for enhancing the electrochemical performance of Zn‐S batteries. By leveraging the unique nano reactor architecture inherent in these carbon spheres, which serve both as reaction templates and as structural confinements. The optimized hollow carbon spheres, with their hollow interiors offering ample space for sulfur accommodation and their hierarchical pore network—including both micropores and mesopores—ensured rapid ion diffusion and uniform distribution of reaction products. Such a configuration not only improves the electrical conductivity by facilitating efficient electron transport pathways but also minimizes the detrimental effects of sulfur and ZnS particle aggregation. Specifically, the optimized HC‐4 exhibited a remarkable capacity retention rate with a decay of only 0.068% per cycle over 500 cycles and a reversible capacity of 463 mAh g^−1^ at high current density.

Finite element simulations further revealed the role of the hierarchical porous design in alleviating stress during cycling, effectively mitigating the structural degradation caused by sulfur volume expansion. Electrochemical and in situ characterizations demonstrated the superior interfacial stability and reaction kinetics achieved with HC‐4.

Taken together, the integrated approach of enhancing conductivity through optimized carbon architectures, limiting ZnS growth via effective nanoconfinement, and relieving mechanical stress by fine‐tuning the shell thickness and pore distribution not only offers an interesting perspective on sulfur cathode design but also assists in the development of high‐performance, durable Zn‐S batteries.

## Conflict of Interest

The authors declare no conflict of interest.

## Supporting information



Supporting Information

## Data Availability

The data that support the findings of this study are available in the supplementary material of this article.

## References

[1] M. Du , P. Geng , J. Shi , H. Xu , W. Feng , H. Pang , Inorg. Chem. 2024, 63, 10823. 38803192 10.1021/acs.inorgchem.4c01553

[2] a) M. Du , J. Shi , P. Geng , W. Zhou , X. Zhang , S. Zhang , H. Pang , Mater. Today Chem. 2024, 41, 102289;

[3] M. Du , P. Geng , C. Pei , X. Jiang , Y. Shan , W. Hu , L. Ni , H. Pang , Angew. Chem., Int. Ed. 2022, 61, 202209350. 10.1002/anie.202209350 36006780

[4] M. Du , J. Shi , Y. Shi , G. Zhang , Y. Yan , P. Geng , Z. Tian , H. Pang , Chem. Sci. 2024, 15, 9775. 38939152 10.1039/d4sc01628a PMC11206441

[5] P. Chen , T. Wang , D. He , T. Shi , M. Chen , K. Fang , H. Lin , J. Wang , C. Wang , H. Pang , Angew. Chem., Int. Ed. 2023, 62, 202311693. 10.1002/anie.202311693 37672488

[6] B. Sun , D. Wang , Y. Jiang , R. Wang , L. Lyu , G. Diao , W. Zhang , H. Pang , Adv. Mater. 2024, 36, 2415633. 10.1002/adma.202415633 39501988

[7] Y. Jiang , M. Du , P. Geng , B. Sun , R. Zhu , H. Pang , J. Colloid Interface Sci. 2024, 664, 617. 38490037 10.1016/j.jcis.2024.03.015

[8] H. Xu , Y. Xiang , X. Xu , Y. Liang , Y. Li , Y. Qi , M. Xu , Adv. Funct. Mater. 2024, 34, 2403663.

[9] a) J. Mao , J. Iocozzia , J. Huang , K. Meng , Y. Lai , Z. Lin , Energy Environ. Sci. 2018, 11, 772;

[10] a) H. Zhang , Z. Shang , G. Luo , S. Jiao , R. Cao , Q. Chen , K. Lu , ACS Nano 2022, 16, 7344; 34889091 10.1021/acsnano.1c08645

[11] M. Wang , H. Zhang , T. Ding , F. Wu , L. Fu , B. Song , P. Cao , K. Lu , Sci. China Chem. 2024, 67, 1531.

[12] T. Zhou , H. Wan , M. Liu , Q. Wu , Z. Fan , Y. Zhu , Mater. Today Energy 2022, 27, 101025.

[13] Y. Zhao , D. Wang , X. Li , Q. Yang , Y. Guo , F. Mo , Q. Li , C. Peng , H. Li , C. Zhi , Adv. Mater. 2020, 32, 2003070. 10.1002/adma.202003070 32596928

[14] X. Zuo , M. Zhen , D. Liu , L. Fu , Y. Qiu , H. Liu , Y. Zhang , Adv. Funct. Mater. 2024, 34, 2405486.

[15] a) G. Zhou , Y. Zhao , A. Manthiram , Adv. Energy Mater. 2015, 5, 1402263;

[16] L. Hu , Y. Chen , Y. Chen , L. Liu , S. Liang , N. Zhou , T. Ding , L. Jiang , L. Wang , X. Liang , K. Hu , Mater. Lett. 2024, 357, 135691.

[17] X. Zhao , Y. Li , Q. Gong , Y. Huang , M. Gong , K. Du , Y. Guo , J. Bai , J. Gan , M. Zhao , Y. Zhao , D. Zhuang , Carbon 2021, 183, 158.

[18] Y. Tang , S. Zheng , S. Cao , F. Yang , X. Guo , S. Zhang , H. Xue , H. Pang , J. Colloid Interface Sci. 2022, 626, 1062. 35839675 10.1016/j.jcis.2022.07.034

[19] S. A. Abbas , M. Forghani , S. Anh , S. W. Donne , K.‐D. Jung , Energy Storage Mater. 2020, 24, 550.

[20] Y. Tang , Y. Shi , Y. Su , S. Cao , J. Hu , H. Zhou , Y. Sun , Z. Liu , S. Zhang , H. Xue , H. Pang , Adv. Sci. 2024, 11, 2403802. 10.1002/advs.202403802 PMC11497006 39140249

[21] L. Peng , C.‐T. Hung , S. Wang , X. Zhang , X. Zhu , Z. Zhao , C. Wang , Y. Tang , W. Li , D. Zhao , J. Am. Chem. Soc. 2019, 141, 7073. 30964289 10.1021/jacs.9b02091

[22] R. Wang , B. Sun , Y. Dong , W. Zhang , Q. Wu , F. Guo , C. Li , W. Li , M. Chen , J. Colloid Interface Sci. 2025, 683, 499. 39700559 10.1016/j.jcis.2024.12.080

[23] L. Ni , J. Gu , X. Jiang , H. Xu , Z. Wu , Y. Wu , Y. Liu , J. Xie , Y. Wei , G. Diao , Angew. Chem., Int. Ed. 2023, 62, 202306528. 10.1002/anie.202306528 37464580

[24] Y. Guo , X. Zhu , J. Zhang , T. Zhang , Z. Wang , M. Shan , F. Wang , C. C. Cao , G. Xu , M. Zhu , Angew. Chem., Int. Ed. 2024, 64, 202422047. 10.1002/anie.202422047 39601202

[25] G. Yang , Q. Zhang , C. He , Z. Gong , Z. Liu , J. Song , S. Jiang , J. Han , H. Yang , X. Li , Z. Pei , S. He , Angew. Chem., Int. Ed. 2025, 137, 202421230. 10.1002/anie.202421230 39741110

[26] M. Yang , Y. Wang , D. Ma , J. Zhu , H. Mi , Z. Zhang , B. Wu , L. Zeng , M. Chen , J. Chen , P. Zhang , Angew. Chem., Int. Ed. 2023, 62, 202304400. 10.1002/anie.202304400 37158757

[27] L. Wang , B. Zhang , W. Zhou , Z. Zhao , X. Liu , R. Zhao , Z. Sun , H. Li , X. Wang , T. Zhang , H. Jin , W. Li , A. Elzatahry , Y. Hassan , H. J. Fan , D. Zhao , D. Chao , J. Am. Chem. Soc. 2024, 146, 6199. 38394360 10.1021/jacs.3c14019

[28] Y. Wang , B. Liang , J. Zhu , G. Li , Q. Li , R. Ye , J. Fan , C. Zhi , Angew. Chem., Int. Ed. 2023, 62, 202302583. 10.1002/anie.202302583 37000437

[29] P. Hei , Y. Sai , C. Liu , W. Li , J. Wang , X. Sun , Y. Song , X. Liu , Angew. Chem., Int. Ed. 2024, 63, 202316082. 10.1002/anie.202316082 38196064

[30] W. Wu , S. Wang , L. Lin , H.‐Y. Shi , X. Sun , Energy Environ. Sci. 2023, 16, 4326.

[31] S. Zhao , X. Wu , J. Zhang , C. Li , Z. Cui , W. Hu , R. Ma , C. Li , J. Energy Chem. 2024, 95, 325

