# Supplementary material for: Nanoreactor‐Driven Uniform Nano ZnS Deposition in Tunable Porous Carbon Spheres for High‐Performance Zn‐S Batteries
Source: Adv Sci (Weinh). 2025 May 8;12(28):2505218. doi: 10.1002/advs.202505218 (PMC12302613; doi:10.1002/advs.202505218)
Supplement: Supplementary file 1 — Supporting Information [file ADVS-12-2505218-s001.docx]

**Supporting Information**

**Nanoreactor-Driven Uniform Nano ZnS Deposition in Tunable Porous Carbon Spheres for High-Performance Zn-S Batteries**

Yuxuan Jiang, ^[a]^ Bingxin Sun, ^[a]^ Dan Wang, ^[a]^ Yan Yan, ^[a]^ Mohsen Shakouri, ^[b]^ Han-Yi Chen, ^[c]^ Wang Zhang, ^[a]^ Rongmei Zhu*^[a]^ and Huan Pang*^[a,d]^

[a] Y.X. Jiang, B.X. Sun, D. Wang, Y. Yan, Prof. W. Zhang, Prof. R.M. Zhu, and Prof. H. Pang
School of Chemistry and Chemical Engineering
Yangzhou University
Yangzhou, Jiangsu, 225009, P. R. China
E-mail: panghuan@yzu.edu.cn, huanpangchem@hotmail.com

[b] Dr. M. Shakouri
Canadian Light Source
University of Saskatchewan
Saskatoon, Saskatchewan, S7N 2V3, Canada

[c] Dr. H.Y. Chen
Department of Materials Science and Engineering
National Tsing Hua University
101, Sec. 2, Kuang-Fu Road, Hsinchu 300044, Taiwan

[d] Prof. H. Pang
State Key Laboratory of Coordination Chemistry
Nanjing University
Nanjing, 210093, P. R. China

**Contents**

[1. Experimental section 5](#_Toc195712634)

[1.1 Chemicals and reagents 5](#_Toc195712635)

[1.2 Materials synthesis 5](#_Toc195712636)

[1.2.1 Synthesis and coating of silicon dioxide spheres: 5](#_Toc195712637)

[1.2.2 Preparation of SiO_2_@C 6](#_Toc195712638)

[1.2.3 Preparation of HCs 6](#_Toc195712639)

[1.3 Preparation of cathode 6](#_Toc195712640)

[1.4 Materials Characterization 7](#_Toc195712641)

[1.5 Battery assembling and test: 8](#_Toc195712642)

[2. Supplementary images and table 10](#_Toc195712643)

[**Figure S1 TEM images with low magnification (the scale bars in the graphs are 100 nm).** 10](#_Toc195712644)

[Figure S2 XRD patterns of HCs. 11](#_Toc195712645)

[Figure S3 Full XPS spectra of HCs. 12](#_Toc195712646)

[Figure S4 High-resolution XPS spectra of C in HCs. 13](#_Toc195712647)

[Figure S5 High-resolution XPS spectra of N in HCs. 14](#_Toc195712648)

[Figure S6 Contour plots of CV curves for the first three turns with different HC cathode. 15](#_Toc195712649)

[Figure S7 CV curves for the first three turns with different HC cathode. 16](#_Toc195712650)

[Figure S8 Contour plots of CV curves for different HC cathodes at different scanning rates. 17](#_Toc195712651)

[Figure S9 CV curves for different HC cathodes at different scanning rates. 18](#_Toc195712652)

[Figure S10 Rate performance of different HC cathodes. 19](#_Toc195712653)

[Figure S11 GCD curves of different HC cathodes. 20](#_Toc195712654)

[Figure S12 GCD curve of the sulfur-free cathode battery. 21](#_Toc195712655)

[**Figure S13 Equivalent fitting circuit model of batteries.** 22](#_Toc195712656)

[Figure S14 Impedance changes of HC-1 during discharge. 23](#_Toc195712657)

[Figure S15 DRT curves of HC-1 during the discharge process. 24](#_Toc195712658)

[Figure S16 Contour plot of DRT curves of HC-1 during charging process. 25](#_Toc195712659)

[**Figure. S17 Ex-situ XPS spectra at different states of charge and discharge.** 26](#_Toc195712660)

[**Figure S18 Practical applications of zinc-sulfur batteries.** 27](#_Toc195712661)

[Figure S19 GCD curves of pouch cell after the first cycle and 50 cycles. 28](#_Toc195712662)

[**Figure S20 Elemental analysis of HCs.** 29](#_Toc195712663)

[**Figure S21 Specific surface area comparison before and after sulfur loading.** 30](#_Toc195712664)

[**Figure S22 Photograph of Swagelok cell.** 31](#_Toc195712665)

[**Table S1 Performance comparison.** 32](#_Toc195712666)

[**Table S2 Inside Volume Ratio and Shell Volume Ratio of HCs.** 33](#_Toc195712667)

[Reference 34](#_Toc195712668)

# 1. Experimental section

## 1.1 Chemicals and reagents

Anhydrous ethanol, NH_3_·H_2_O, tetraethyl orthosilicate (TEOS), tetrapropyl orthosilicate (TPOS), sodium hydroxide, formaldehyde and resorcinol were obtained from Aladdin (China). Super P and N-Methyl pyrrolidone (NMP) were purchased from Shanghai Chemical Reagents Company (Shanghai, China). All the reagents used are analytically pure.

## 1.2 Materials synthesis

### 1.2.1 Synthesis and coating of silicon dioxide spheres:

Add the silicon source to a solution containing ethanol (50 mL), H_2_O (10 mL), and NH_3_·H_2_O (2 mL) at room temperature with stirring. After stirring for 30 minutes, add resorcinol (0.400 g) and formaldehyde (0.560 mL) to the solution, stir for 24 hours, centrifuge the precipitate at a rate of 6000 r/min, wash with water and ethanol, and dry at 60 ℃ for about 12 hours to obtain the precipitate SiO_2_@SiO_2_/RF composite materials

Preparation of series using TEOS/TPOS with different molar ratios (fixed total amount of 10 mmol) as silicon source SiO_2_@SiO_2_/RF composite material, other conditions remain unchanged.

### 1.2.2 Preparation of SiO_2_@C

The dried SiO_2_@SiO_2_/RF composites were heated from room temperature to 700 °C in a tube furnace under nitrogen atmosphere at an increasing rate of 1 °C/min to 700 °C for 5 h under nitrogen atmosphere and then cooled to room temperature. The obtained samples were named as SiO_2_@C.

### 1.2.3 Preparation of HCs

The carbonation products were dispersed in 2 M sodium hydroxide solution for 48 h for etching to remove silica. The products were recovered by centrifugation, washed to neutrality with water and ethanol, and dried under vacuum at 60 °C for 12 h. HCs were obtained, named HC-x (x is the ordinal number of the silica spheres employing different TEOS/TPOS molar ratios). In the synthesis process, the ratio of TEOS to TPOS was systematically adjusted to control the structural characteristics of the resulting carbon spheres. Specifically, samples prepared with pure TEOS, TEOS:TPOS ratios of 4:1, 1:1, and 1:4, as well as pure TPOS, were designated as HC-1, HC-2, HC-3, HC-4, and HC-5, respectively.

## 1.3 Preparation of cathode

HCs were first prepared to used as the activated material of Zn-S cells through melt-diffusion method (HCs and sublimation of sulfur mixed uniformly into the hydrothermal reaction kettle sealed, warmed to 155 ℃ insulation 12h). Figure S20 illustrates the uniform loading of S in HCs. The specific surface area of the host showed a significant decrease after sulfur loading (Figure S21), which indicates the successful loading of sulfur in the carbon spheres. For the preparation of cathode, HCs/S, Super P, and NMP solution containing 5 wt.% PVDF (8:1:1 by weight) were milled together to form uniform slurry. Then, the slurry was coated on the carbon paper and dried in vacuum at 60 ℃ for 12 h.

## 1.4 Materials Characterization

The Powder X-ray diffraction (PXRD) patterns were performed by Bruker AXS D8 advance with Cu Kα radiation of 40 kV (λ=1.5418 Å). Scanning electron microscopy (SEM) images were obtained by Zeiss-Supra 55 microscope. Transmission electron microscopy (TEM) was recorded using Tecnai G2 F30 S-TWIN at an acceleration voltage of 300 kV. Raman spectroscopy was obtained by using Renishaw InVia Reflex (514 nm laser). Nitrogen sorption isotherms were carried out using a BELSORP-mini (BEL, Japan). The specific surface area (SSA) was analyzed by Multipoint Brunauer-Emmett-Teller (BET) technique. X-ray photoelectron spectroscopy (XPS) analysis was carried out using a Thermo Scientific ESCALAB 250Xi X-ray photoelectron spectrometer with Al Kα radiation of 1486.6 eV as the excitation source. The survey thickness is 2-3 nm. The concentration variations in these solutions were detected by the UV-vis spectroscopy.

## 1.5 Battery assembling and test:

The CR 2032-type coin cells were fabricated using the working electrode, zinc foil as anode electrode, glass fiber as the separator. The electrolyte is composed of 2M ZnSO_4_ and 0.05M ZnI_2_ (100 μL per cell). The GCD tests were estimated in the voltage window of 0.1-1.5 V. The rate capability was also tested by varying the current density from 0.1 A/g to 5 A/g on a battery measurement system (CT2001A, Wuhan Land, China) at room temperature. CV and EIS curves were measured on an electrochemical workstation (CHI660E, Chenhua, Shanghai, China). CV curves was performed from 1.5 V to 0.1 V at a scanning rate of 0.1 mV s^-1^, and the frequency of EIS was performed form 100 kHz to 0.01 Hz at open-circuit potential. For In situ EIS measurements, Zn-S cells were discharged in galvanostatic mode at 0.1 A/g for 10 min, and then the cell was rest for 10 min to reach the quasi-open circuit voltage (QOCV). The EIS test is performed when the QOCV is slightly below the target voltage. Electrodes for ex-situ XRD analysis were obtained from Swagelok cells (Figure S22). The cells were discharged under a constant current until the target voltage was reached, at which point the cells were promptly disassembled to extract the cathode. The removed cathode was then dried at room temperature until completely moisture-free and subsequently subjected to X-ray diffraction analysis over an angular range of 20° to 60° at a scanning speed of 5°/min.

# 2. Supplementary images and table


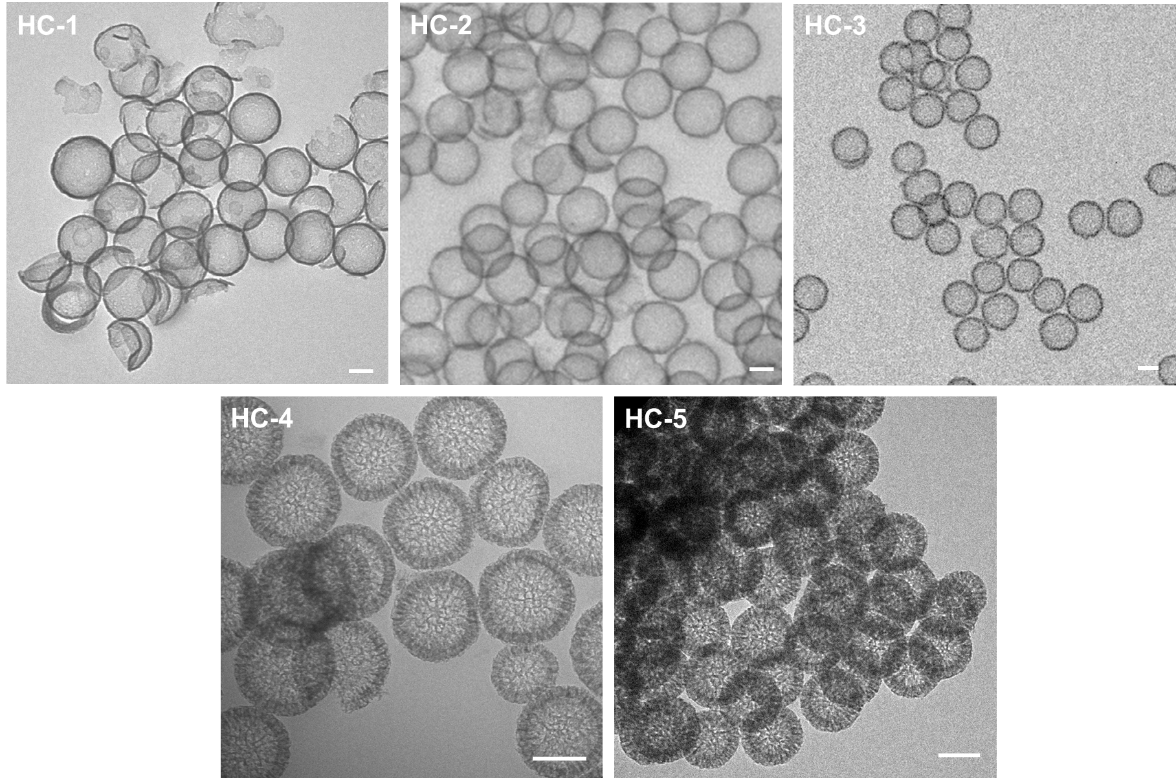


**Figure S1 TEM images with low magnification (the scale bars in the graphs are 100 nm).**


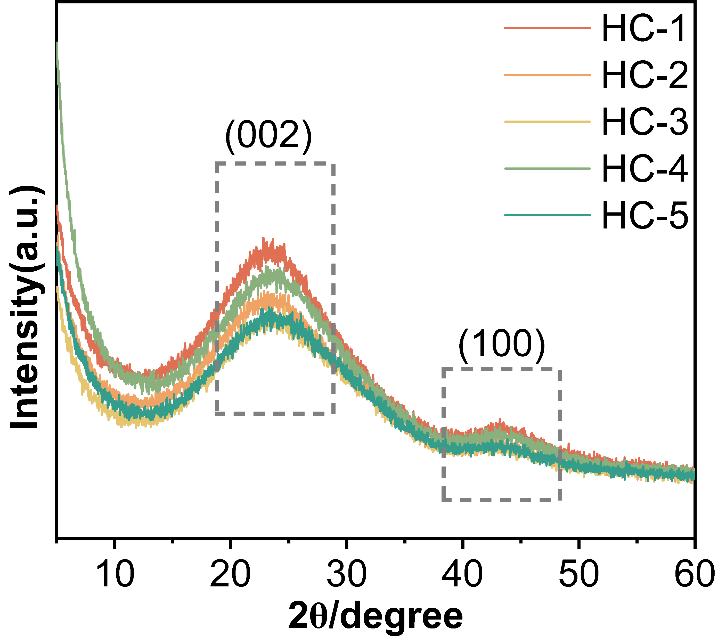


## Figure S2 XRD patterns of HCs.


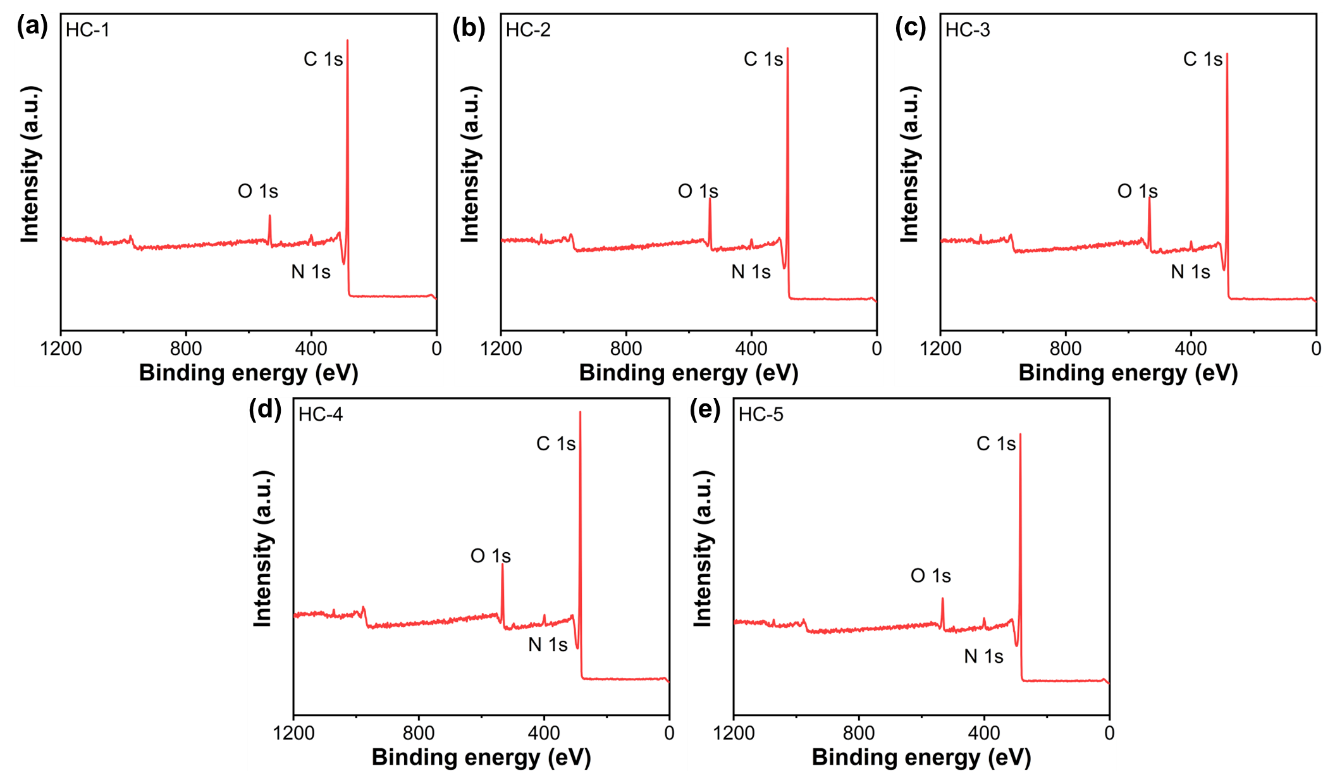


## Figure S3 Full XPS spectra of HCs.


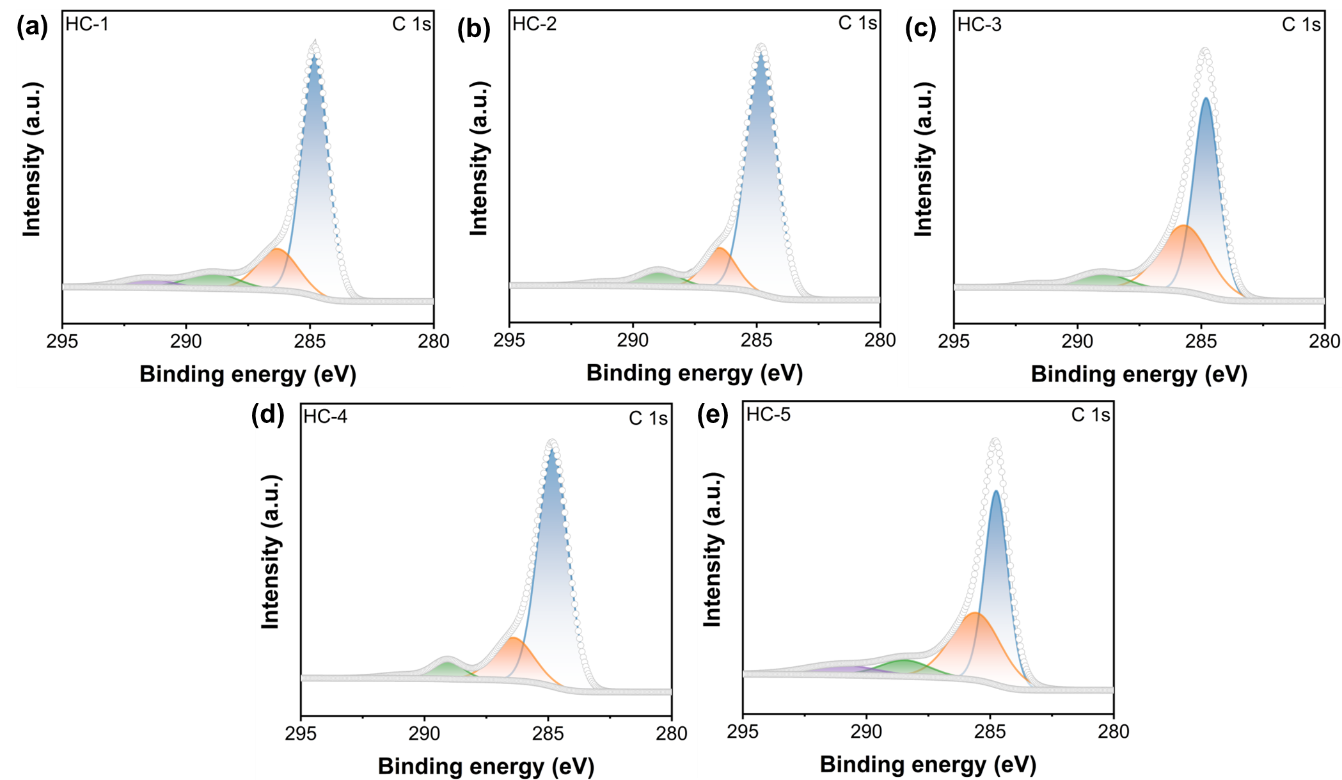


## Figure S4 High-resolution XPS spectra of C in HCs.


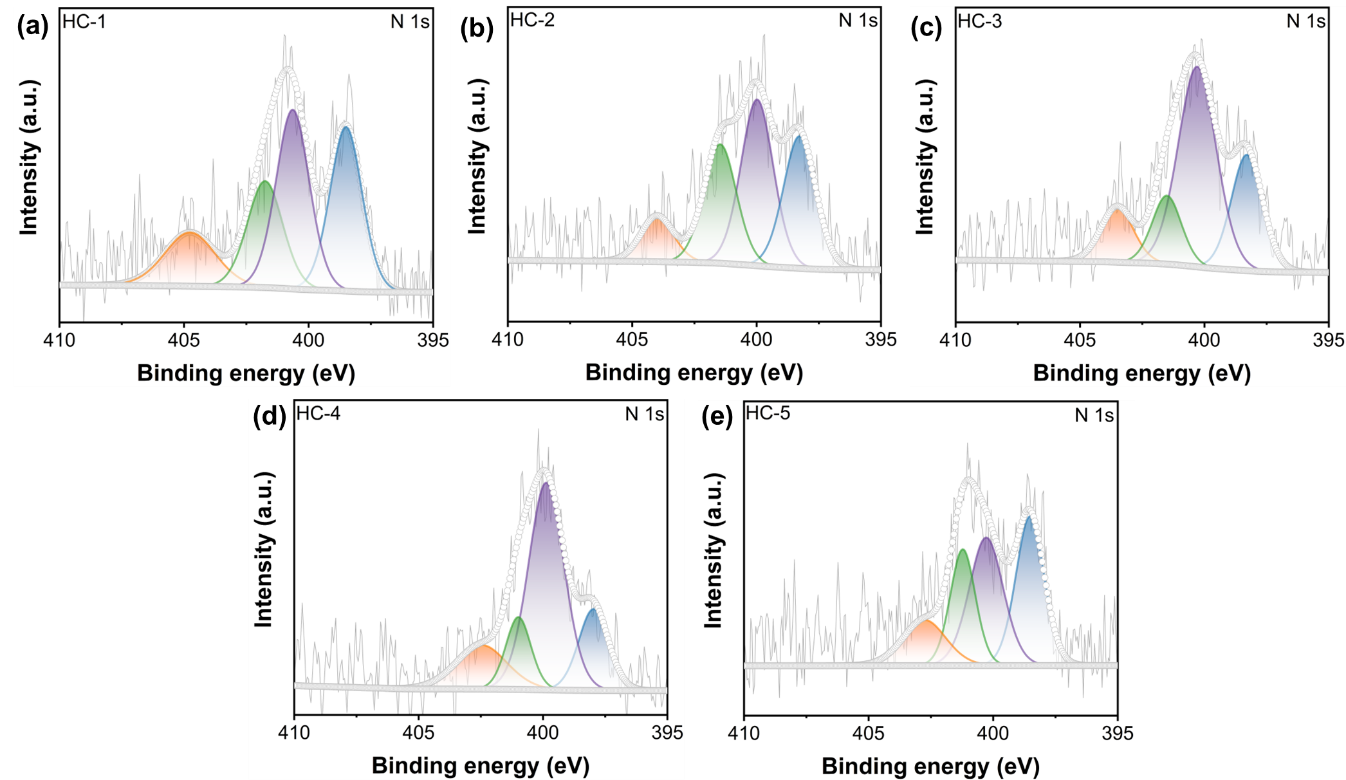


## Figure S5 High-resolution XPS spectra of N in HCs.


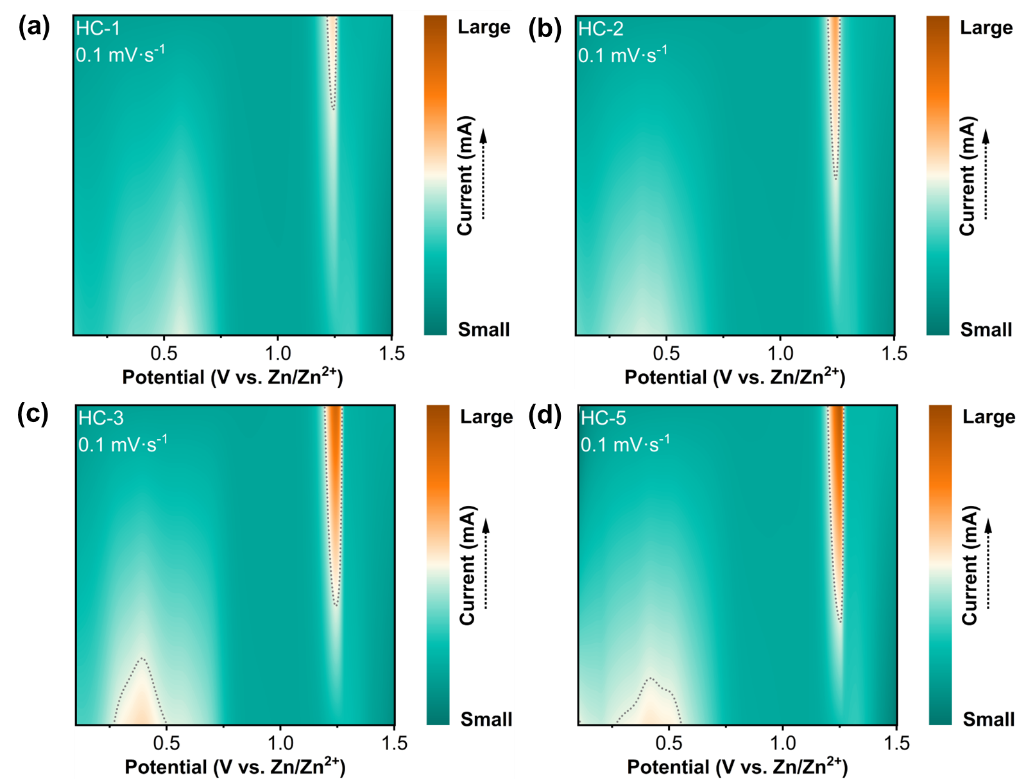


## Figure S6 Contour plots of CV curves for the first three turns with different HC cathode.


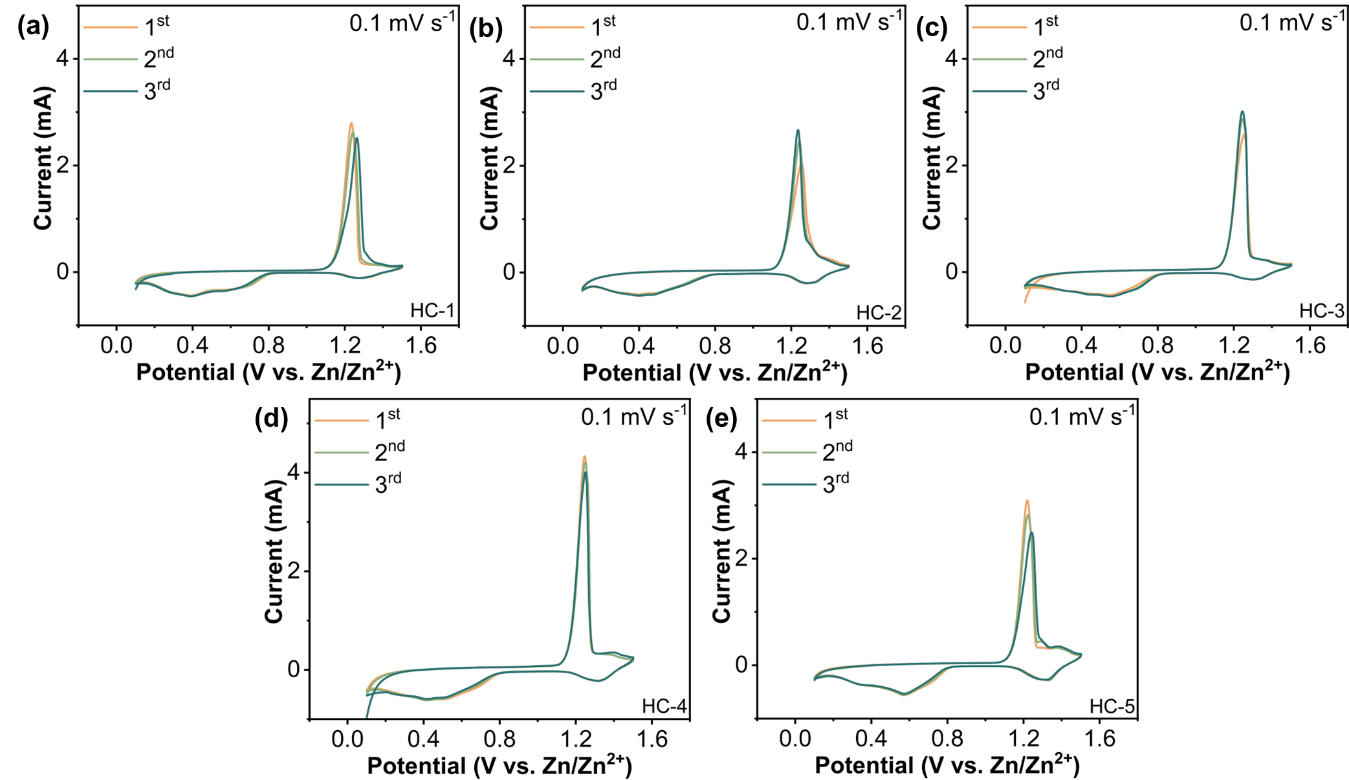


## Figure S7 CV curves for the first three turns with different HC cathode.


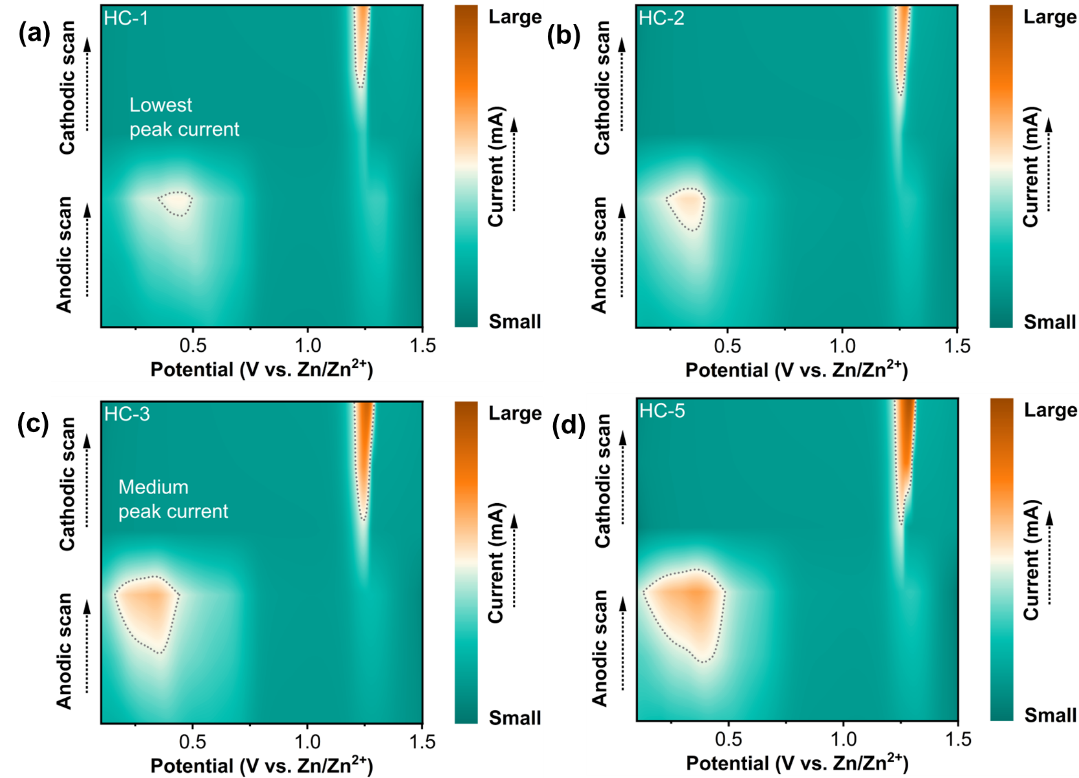


## Figure S8 Contour plots of CV curves for different HC cathodes at different scanning rates.


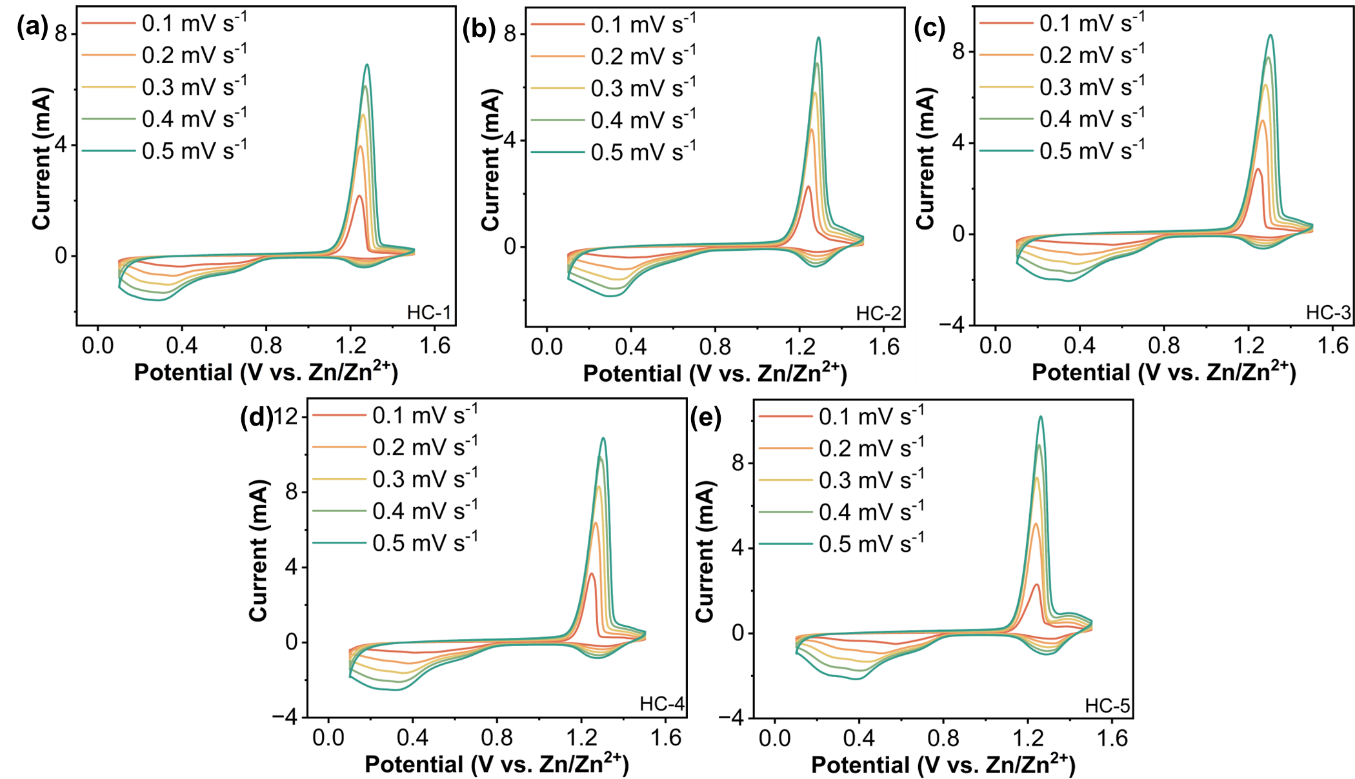


## Figure S9 CV curves for different HC cathodes at different scanning rates.


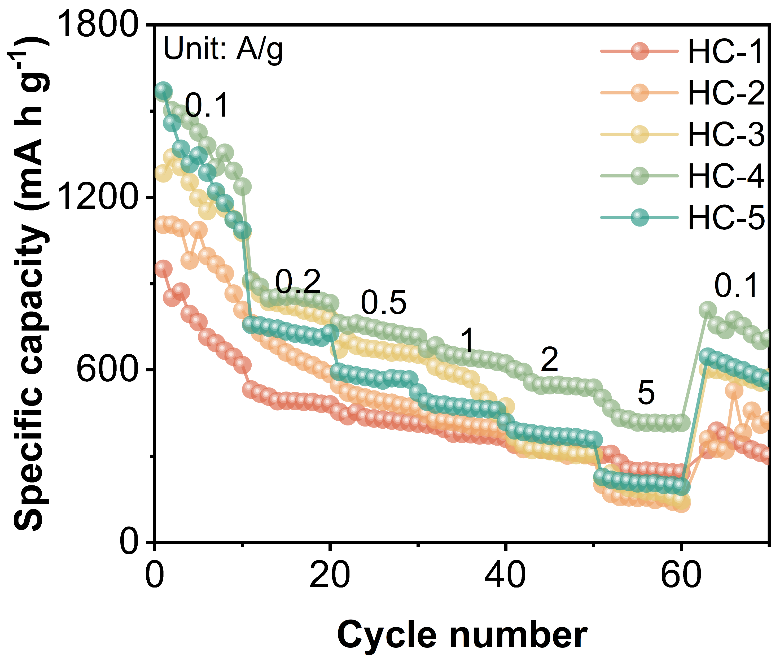


## Figure S10 Rate performance of different HC cathodes.


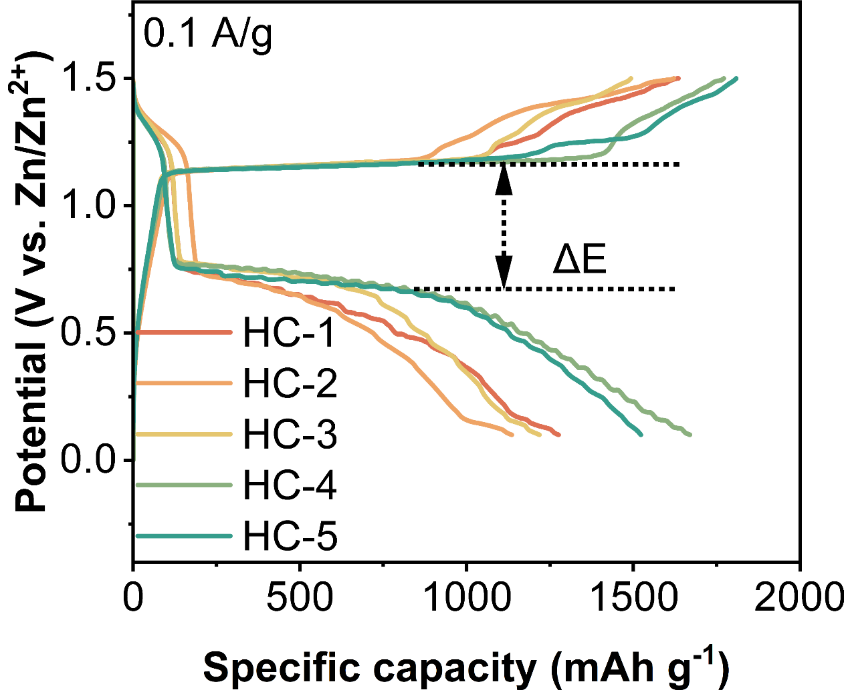


## Figure S11 GCD curves of different HC cathodes.


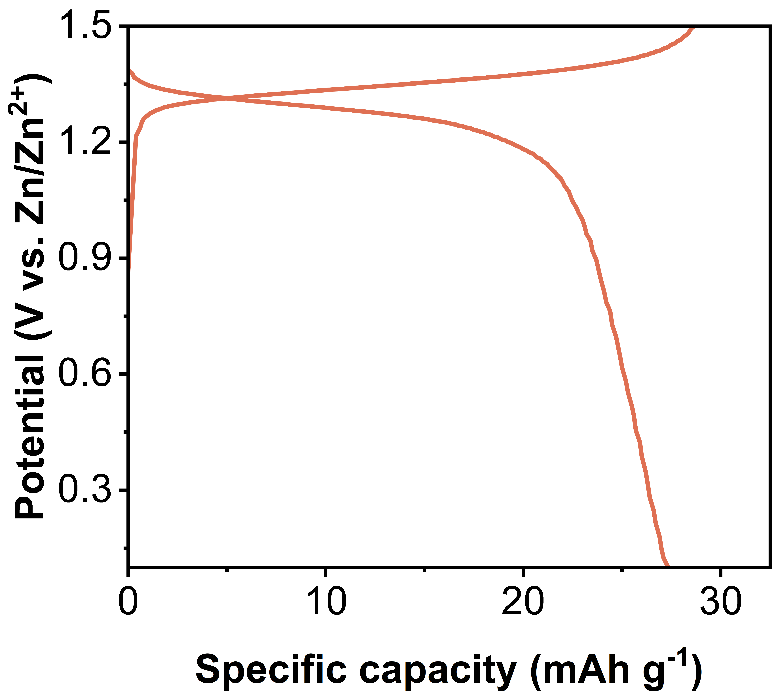


## Figure S12 GCD curve of the sulfur-free cathode battery.


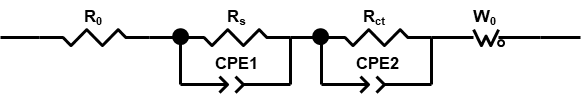


**Figure S13 Equivalent fitting circuit model of batteries.**


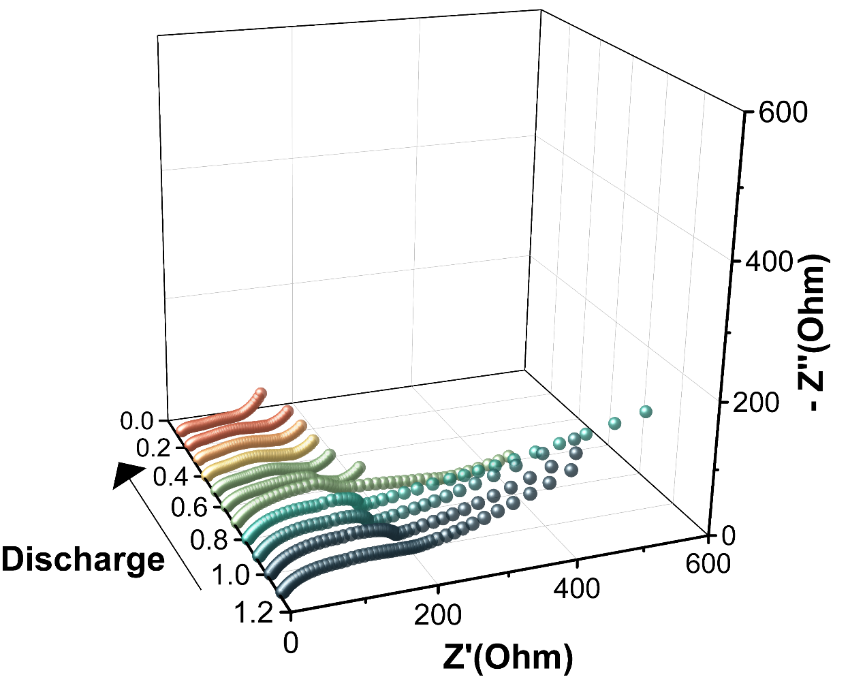


## Figure S14 Impedance changes of HC-1 during discharge.


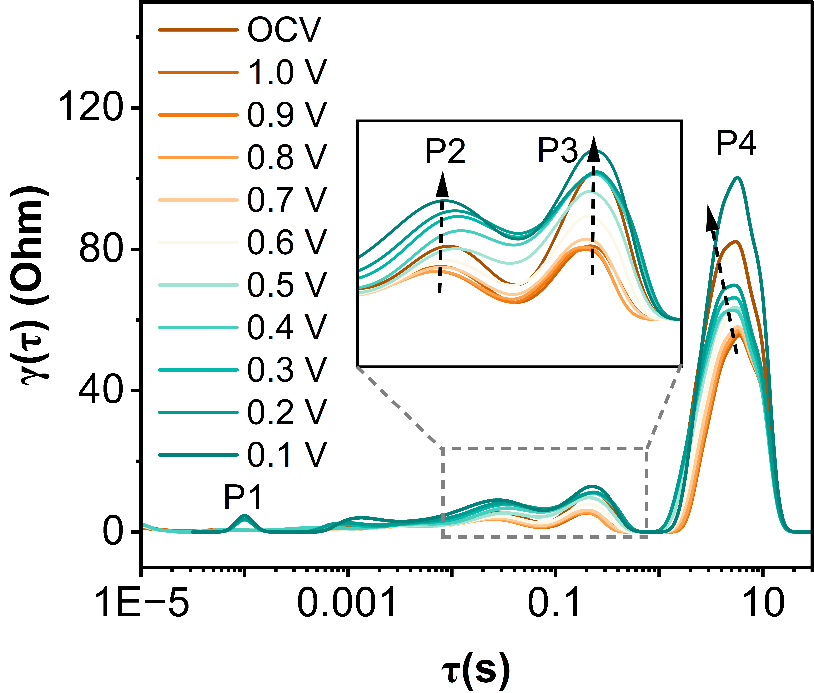


## Figure S15 DRT curves of HC-1 during the discharge process.


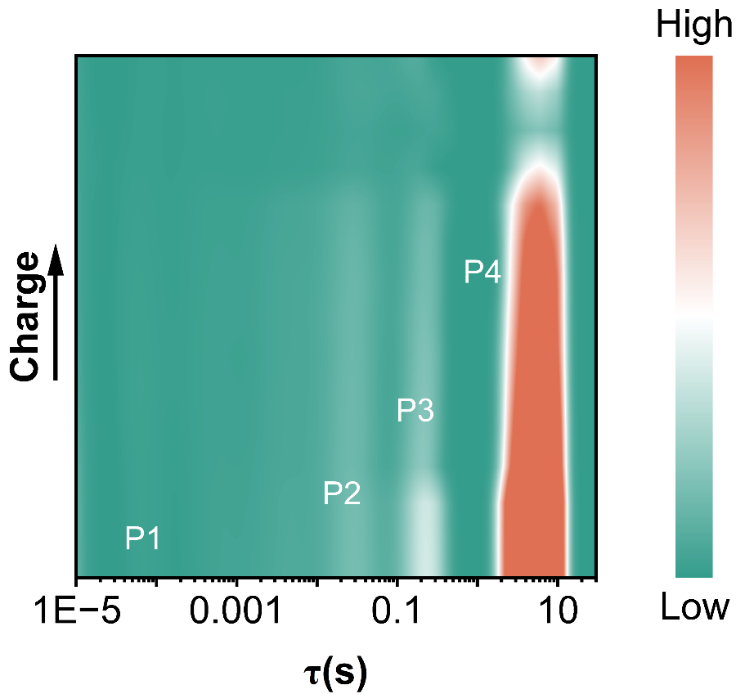


## Figure S16 Contour plot of DRT curves of HC-1 during charging process.


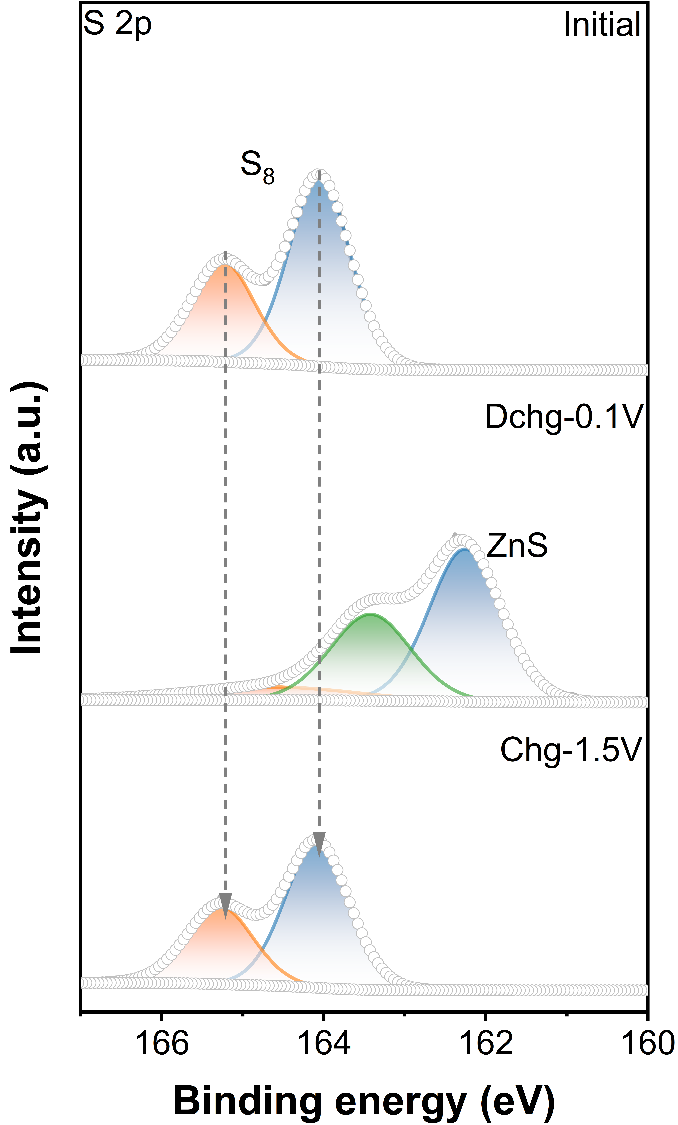


**Figure. S17 Ex-situ XPS spectra at different states of charge and discharge.**


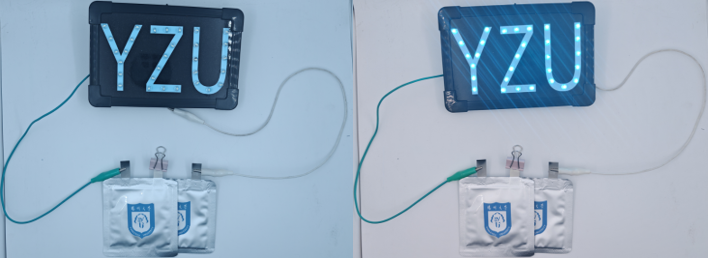


**Figure S18 Practical applications of zinc-sulfur batteries.**


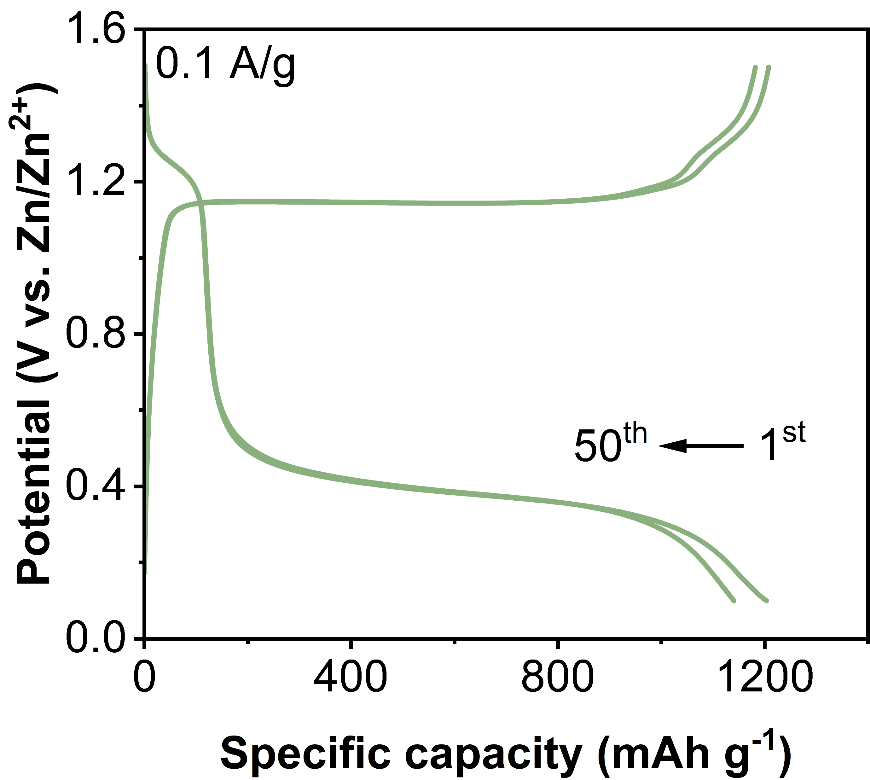


## Figure S19 GCD curves of pouch cell after the first cycle and 50 cycles.

**Figure S20 Elemental analysis of HCs.**


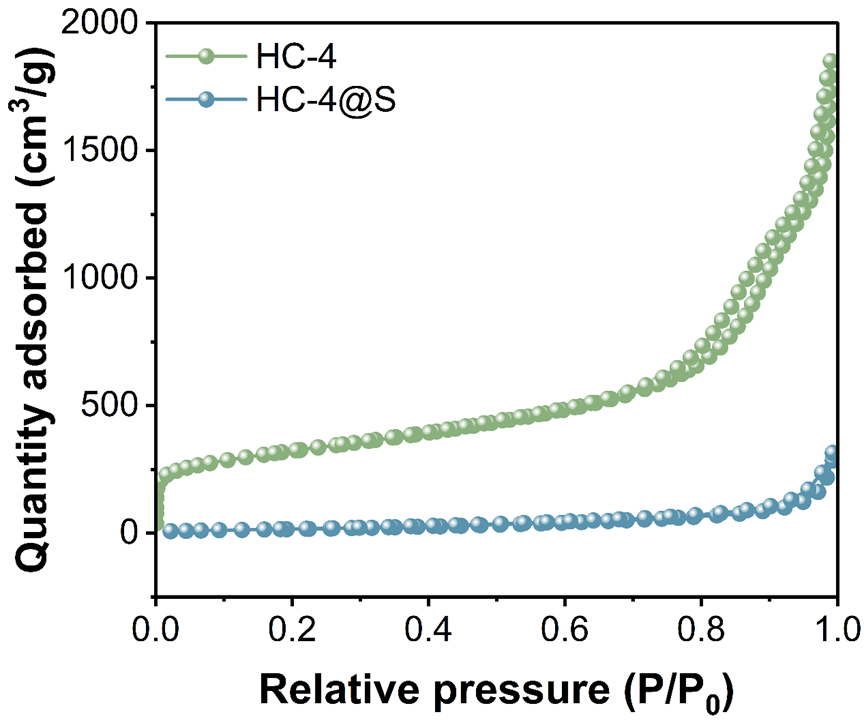


**Figure S21 Specific surface area comparison before and after sulfur loading.**


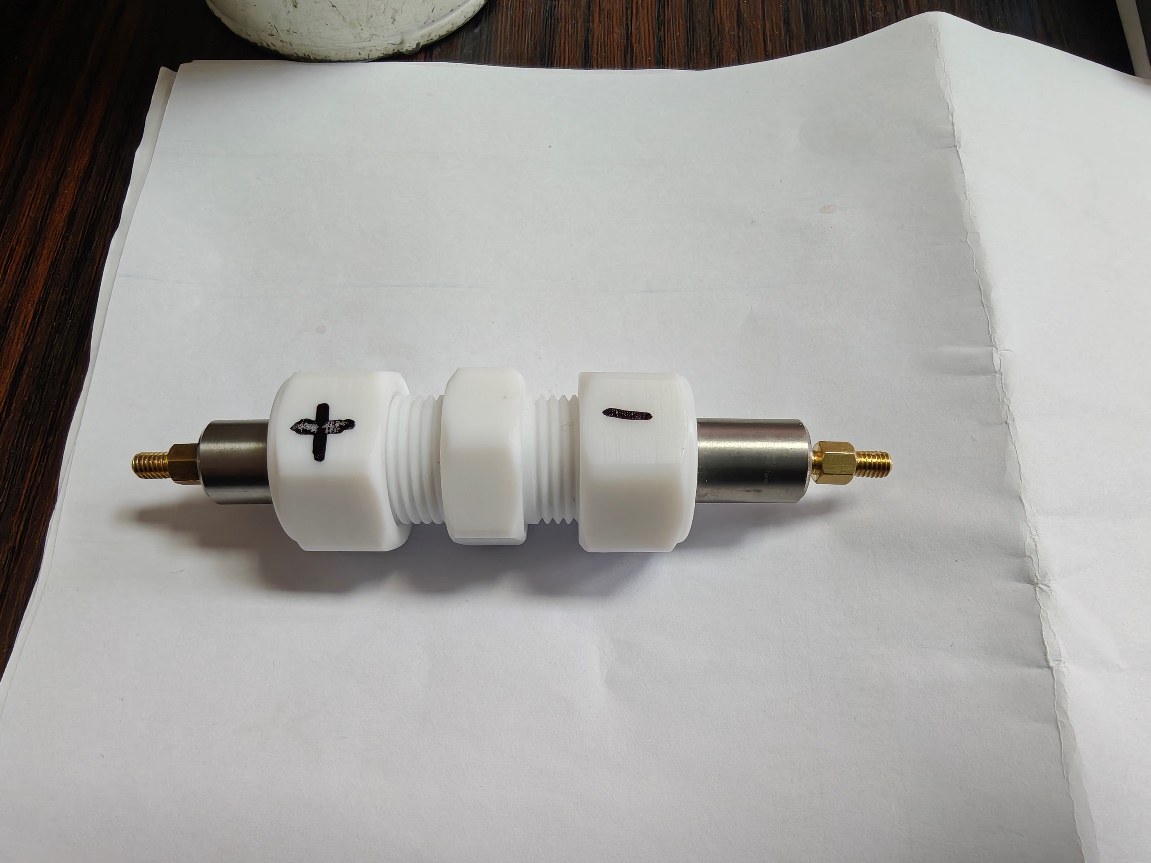


**Figure S22 Photograph of Swagelok cell.**

**Table S1 Performance comparison.**

| Cathode | Electrolyte | Current density [A/g] | Specific capacity  [mAh/g] | Rate  [A/g]/ [mAh/g] | Cycle number/Capacity retention (%)/Current density [A g^-1^] | Ref |
| --- | --- | --- | --- | --- | --- | --- |
| S@FeNC/NC/CC | 2 M ZnSO_4_ | 0.2 | 1143 | 2/528 | 300/57/0.5 | [1] |
| CNT/S | 1 M Zn(CH_3_COO)_2_ with PEG-400 | 0.1 | 1079 | 2/335 | 300/73/1 | [2] |
| S@C | 2 M ZnSO_4_ + 0.1 wt% I_2_ | 0.5 | 864 | 4/122 | 400/30/1 | [3] |
| KB/S | 2 M ZnSO_4_ + ZnI + x M TU | 0.1 | 1478 | 2/900 | 300/53/2 | [4] |
| S@CNTs-50 | 1 M Zn(CH_3_COO)_2_ + 0.05 wt% I_2_ | 0.1 | 1105 | 4/407 | 225/37/2 | [5] |
| CMK-3/S | 2 M ZnSO_4_ + 20 mM ZnI_2_ | 0.1 | 1630 | 3/743 | 300/49/2 | [6] |
| AJPC/S | Zn(OTF)_2_/I_2_/DMC | 0.1 | 1167 | 2/218 | 200/48/1 | [7] |
| **HCs@S** | 2 M ZnSO_4_ + 50 mM ZnI_2_ | 0.1 | 1558 | 5/463 | 665/41/2 | **This work** |

**Table S2 Inside Volume Ratio and Shell Volume Ratio of HCs.**

| **Sample** | **d (nm)** | **t (nm)** | **Inside Diameter (d-2t) (nm)** | **Inside Volume Ratio (%)** | **Shell Volume Ratio (%)** |
| --- | --- | --- | --- | --- | --- |
| HC-1 | 197 | 4 | 189 | ≈88.3 | ≈11.7 |
| HC-2 | 183 | 6 | 171 | ≈81.6 | ≈18.4 |
| HC-3 | 153 | 9 | 135 | ≈68.5 | ≈31.5 |
| HC-4 | 128 | 15 | 98 | ≈44.9 | ≈55.1 |
| HC-5 | 115 | 22 | 71 | ≈23.5 | ≈76.5 |

Reference

[1] Zhang, Weiwei, et al. "Bidirectional Atomic Iron Catalysis of Sulfur Redox Conversion in High‐Energy Flexible Zn-S Battery." *Advanced Functional Materials* 33.11 (2023): 2210899.

[2] Zhou, Tiansheng, et al. "Regulating uniform nucleation of ZnS enables low-polarized and high stable aqueous Zn–S batteries." *Materials Today Energy* 27 (2022): 101025.

[3] Cui, Mangwei, et al. "Ultra-High-Capacity and dendrite-free zinc–sulfur conversion batteries based on a low-cost deep eutectic solvent." *ACS Applied Materials & Interfaces* 13.46 (2021): 54981-54989.

[4] Li, Jianbo, et al. "Constructing a raincoat-like protective layer on sulfur cathode for aqueous Zn–S batteries." *Energy Storage Materials* 70 (2024): 103541.

[5] Li, Wei, Kangli Wang, and Kai Jiang. "A low cost aqueous Zn–S battery realizing ultrahigh energy density." *Advanced science* 7.23 (2020): 2000761.

[6] Li, Jianbo, et al. "Rational design of zinc powder anode with high utilization and long cycle life for advanced aqueous Zn–S batteries." *Materials Horizons* 10.7 (2023): 2436-2444.

[7] Patel, Dinesh, et al. "Hybrid electrolyte with biomass-derived carbon host for high-performance aqueous Zn–S battery." *Chemical Engineering Journal* 479 (2024): 147722.
